# Mitochondrial respiratory complex III sustains IL-10 production in activated macrophages and promotes tumor-mediated immune evasion

**DOI:** 10.1126/sciadv.adq7307

**Published:** 2025-01-22

**Authors:** Alessia Zotta, Juliana Toller-Kawahisa, Eva M. Palsson-McDermott, Shane M. O’Carroll, Órlaith C. Henry, Emily A. Day, Anne F. McGettrick, Ross W. Ward, Dylan G. Ryan, Mark A. Watson, Martin D. Brand, Marah C. Runtsch, Kathrin Maitz, Anna Lueger, Julia Kargl, Jan L. Miljkovic, Ed C. Lavelle, Luke A. J. O’Neill

**Affiliations:** ^1^ School of Biochemistry and Immunology, Trinity Biomedical Sciences Institute, Trinity College Dublin, Dublin 2, Ireland.; ^2^ Department of Physiology and Pharmacology, Schulich School of Medicine & Dentistry, University of Western Ontario, London, ON, Canada.; ^3^ Mitochondria Biology Unit, University of Cambridge, Cambridge, UK.; ^4^ Buck Institute for Research on Aging, Novato, CA, USA.; ^5^ Division of Pharmacology, Otto Loewi Research Center, Medical University of Graz, Graz, Austria.

## Abstract

The cytokine interleukin-10 (IL-10) limits the immune response and promotes resolution of acute inflammation. Because of its immunosuppressive effects, IL-10 up-regulation is a common feature of tumor progression and metastasis. Recently, IL-10 regulation has been shown to depend on mitochondria and redox-sensitive signals. We have found that Suppressor of site III_Qo_ Electron Leak 1.2 (S3QEL 1.2), a specific inhibitor of reactive oxygen species (ROS) production from mitochondrial complex III, and myxothiazol, a complex III inhibitor, decrease IL-10 in lipopolysaccharide (LPS)–activated macrophages. IL-10 down-regulation is likely to be mediated by suppression of c-Fos, which is a subunit of activator protein 1 (AP1), a transcription factor required for *IL-10* gene expression. S3QEL 1.2 impairs IL-10 production in vivo after LPS challenge and promotes the survival of mice bearing B16F10 melanoma by lowering tumor growth. Our data identify a link between complex III–dependent ROS generation and IL-10 production in macrophages, the targeting of which could have potential in boosting antitumor immunity.

## Teaser

Mitochondrial respiratory complex III sustains IL-10 production in macrophages and promotes tumor-mediated immune evasion.

## INTRODUCTION

Immune cells undergo metabolic reprogramming during activation (*1*), and the intricacies of the metabolic processes occurring are still being unraveled. Macrophages have been a key focus with profound changes in glycolysis and mitochondrial metabolism occurring after stimulation with activators such as lipopolysaccharide (LPS) from Gram-negative bacteria (*2*).

They produce, among many cytokines, the anti-inflammatory cytokine interleukin-10 (IL-10). Initially found as a T helper 2 (T_H_2)–specific cytokine (*3*), IL-10 is a soluble mediator whose production is shared by innate and adaptive immune cells such as dendritic cells, monocytes, B cells, and regulatory T cells (T_regs_) (*4*). IL-10 can elicit immune suppression by limiting antibody production in B cells and costimulatory molecules in T cells (*5*). Considering the further literature on mitochondrial biology, IL-10 has been shown to decrease reactive oxygen species (ROS) production, drive controlled mitophagy, and promote mitochondrial fitness (*6*). IL-10 deficiency leads to dysfunctional mitochondria and impaired oxidative phosphorylation (OXPHOS) with an increase in glycolysis in macrophages (*6*). More recently, the redox protein thioredoxin has been shown to be necessary for the differentiation of IL-10^+^ regulatory B cells through a mechanism that includes controlling ROS generation (*7*), providing a link between oxidative stress and IL-10.

Mitochondrial respiratory complex III is part of the electron transport chain (ETC), where it oxidizes coenzyme Q and reduces cytochrome c to generate adenosine triphosphate (ATP) during oxidative phosphorylation. It is also the main source of superoxide anion (O_2_^·−^) and subsequent ROS from mitochondria, together with complex I, during forward and reverse electron transport (FET and RET, respectively) (*8*). In humans, mutations in three complex III–specific genes lead to mitochondrial disorders with different onset and clinical manifestations (*9*), making their characterization and treatment a challenge. The contribution of complex III to immune cell function requires further exploration. Complex III has been shown to be essential for hematopoietic stem cell stability by regulating epigenetic changes in histones (*10*) and for the immune suppressive functions of T_regs_ (*11*). Moreover, complex III is required for antigen-specific activation of cytotoxic T cells via ROS (*12*) and for complex I stability and maintenance along the ETC (*13*). The use of complex III–specific inhibitors, such as myxothiazol (MYX), has helped in deciphering the multifaceted nature of complex III. In addition, the small molecule Suppressor of site III_Qo_ Electron Leak (S3QEL) has been developed to stop electron leak from the ETC to oxygen, thus blocking ROS production specifically from complex III (*14*, *15*).

Here, we have examined the role of complex III in macrophages using S3QEL 1.2 and MYX. We have found that inhibition of complex III activity and subsequent ROS generation lead to a decrease in IL-10 production in vitro and in vivo and that the down-regulation of the transcription factor activator protein 1 (AP1) might contribute to this event. Moreover, we show that S3QEL 1.2 treatment decreases melanoma progression and improves the survival of mice in vivo, impairing the production of IL-10 by macrophages in the tumor microenvironment (TME). Our results indicate a role for ROS from complex III in the induction of IL-10 in macrophages, the limiting of which could have utility in the promotion of antitumor immunity.

## RESULTS

### S3QEL 1.2 and MYX block ROS production by affecting OXPHOS in macrophages

**Fig. 1.**
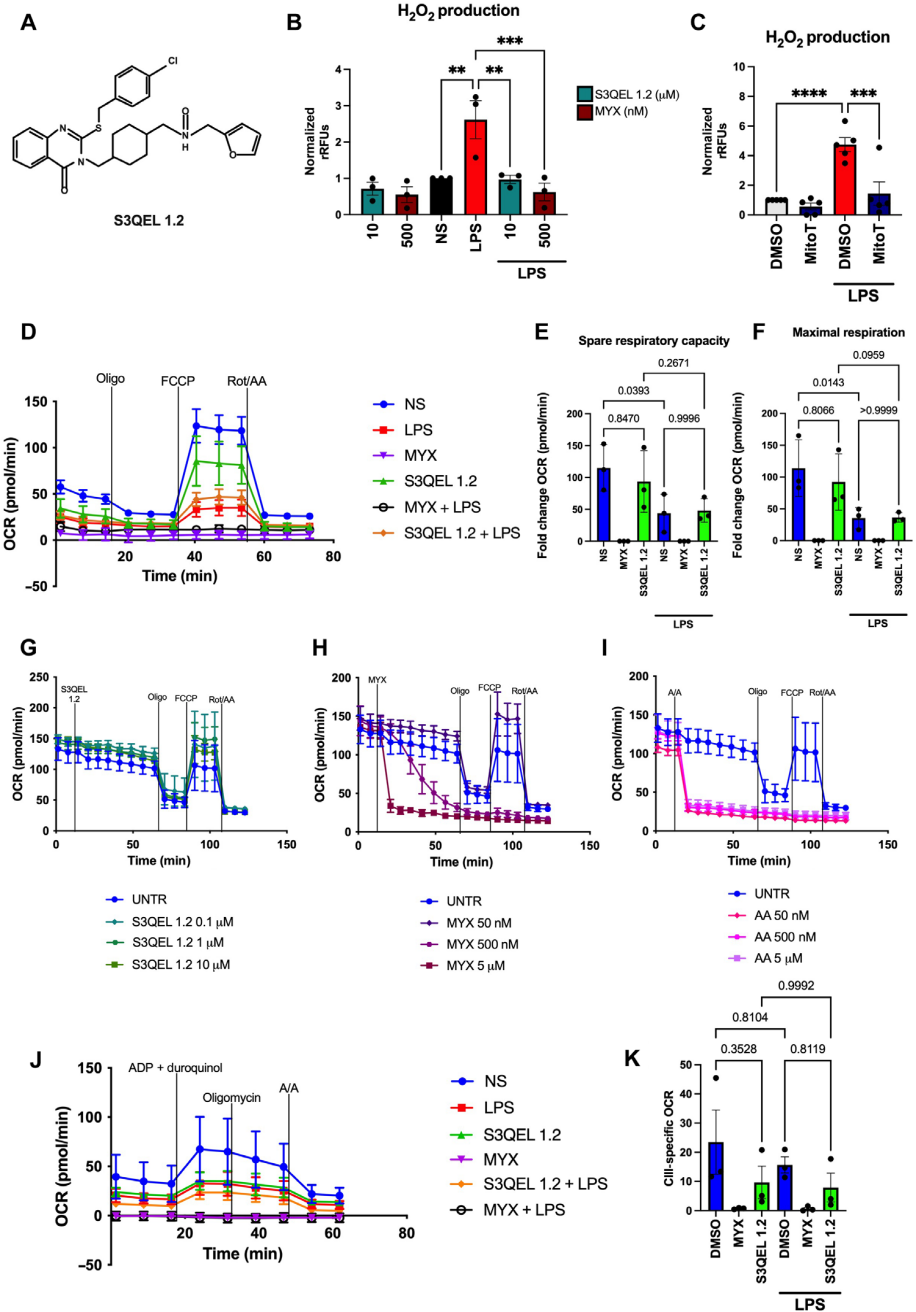
Characterization of complex III inhibitors in macrophages. (**A**) S3QEL 1.2 chemical structure. (**B** and **C**) Rate of relative resorufin fluorescence units (rRFUs) directly proportional to H_2_O_2_ production in the presence of DMSO, S3QEL 1.2 (10 μM), and MYX (500 nM) (B) or DMSO, MitoTempo (5 mM), and LPS (100 ng/ml) (C) for 48 hours in BMDMs. NS, not stimulated. (**D** to **F**) BMDMs were pretreated with DMSO, MYX (500 nM), or S3QEL 1.2 (10 μM) for 3 hours followed by LPS (100 ng/ml) for 24 hours. Mito Stress Test showing OCR of cells after addition of oligo, FCCP, and rotenone (Rot)/AA (D). Maximal respiration (E) and spare respiratory capacity (F) calculated from the OCR values. (**G** to **I**) Mito Stress Test showing OCR of cells after treatment with S3QEL 1.2 (0.1, 1, or 10 μM) (G), MYX (50, 500, or 5000 nM) (H), or AA (50, 500, or 5000 nM) (I) and addition of oligo, FCCP, and rotenone/AA. UNTR, untreated. (**J** to **K**) BMDMs were pretreated with DMSO, MYX (500 nM), or S3QEL 1.2 (10 μM) for 3 hours followed by LPS (100 ng/ml) for 24 hours. Metabolic flux analysis showing complex III (CIII)–specific OCR of cells over time after addition of ADP + duroquinol, oligo, and AA. (K) Quantification of complex III–specific OCR. Data in (B) are means ± SEM for *n* = 3 from three independent experiments. Data in (C) are means ± SEM for *n* = 5 from five independent experiments. Data from (D) to (F) are means ± SEM from three independent experiments, with *n* = 7 to 12 technical replicates for each condition. Data from (G) to (I) are means ± SEM from two independent experiments, with *n* = 9 technical replicates for each condition. Data from (J) to (K) are means ± SEM from three independent experiments, with *n* = 12 technical replicates for each condition. *P* values were calculated using one-way ANOVA for multiple comparisons. Differences were statistically significant at **P < 0.01, ***P < 0.001, and ****P < 0.0001.

The previous literature has described S3QEL 1.2 (Fig. 1A), developed by Brand and colleagues (*15*), as a specific inhibitor of complex III–derived ROS production (*15*), but its effect in immune cells has not been established. Here, we sought to investigate the effects of S3QEL 1.2 on activated bone marrow–derived macrophages (BMDMs) and to compare it to MYX, a specific inhibitor for complex III activity. First, we confirmed that LPS increased ROS production in macrophages by measuring H_2_O_2_ release and demonstrated that S3QEL 1.2 and MYX inhibited this response (Fig. 1B) The same result was obtained with the mitochondria-targeted antioxidant MitoTEMPO at 5 mM, which decreased LPS-induced H_2_O_2_ production (Fig. 1C). We then performed metabolic flux analysis to assess whether complex III inhibitors were perturbing respiration of macrophages. As shown in Fig. 1 (D to F), S3QEL 1.2 was marginally inhibitory against OXPHOS in macrophages at 10 μM with 500 nM MYX abolishing respiration. LPS inhibited OXPHOS as shown previously (*16*). To confirm this, we performed metabolic flux analysis with different concentrations of S3QEL 1.2, MYX, and AA, another complex III inhibitor, injected into BMDMs in real time. Also here, S3QEL 1.2 did not affect respiration at any concentration used (Fig. 1G), while MYX and AA impaired it (Fig. 1, H and I). Moreover, S3QEL 1.2 did not change the activity of complex III, while MYX completely disrupted it (Fig. 1, J and K), as shown in a previous study (*17*). The two inhibitors were also not toxic to the cells, as shown by 3-(4,5-dimethylthiazol-2-yl)-2,5-diphenyltetrazolium (MTT) assay to examine the viability of cells (fig. S1A), and did not change the protein abundance of the ETC complexes (fig. S1B), analyzed by Western blotting. S3QEL 1.2 and MYX also slightly boosted glycolysis (fig. S1, C and D).

### Complex III inhibition provokes specific transcriptional changes in activated macrophages

**Fig. 2.**
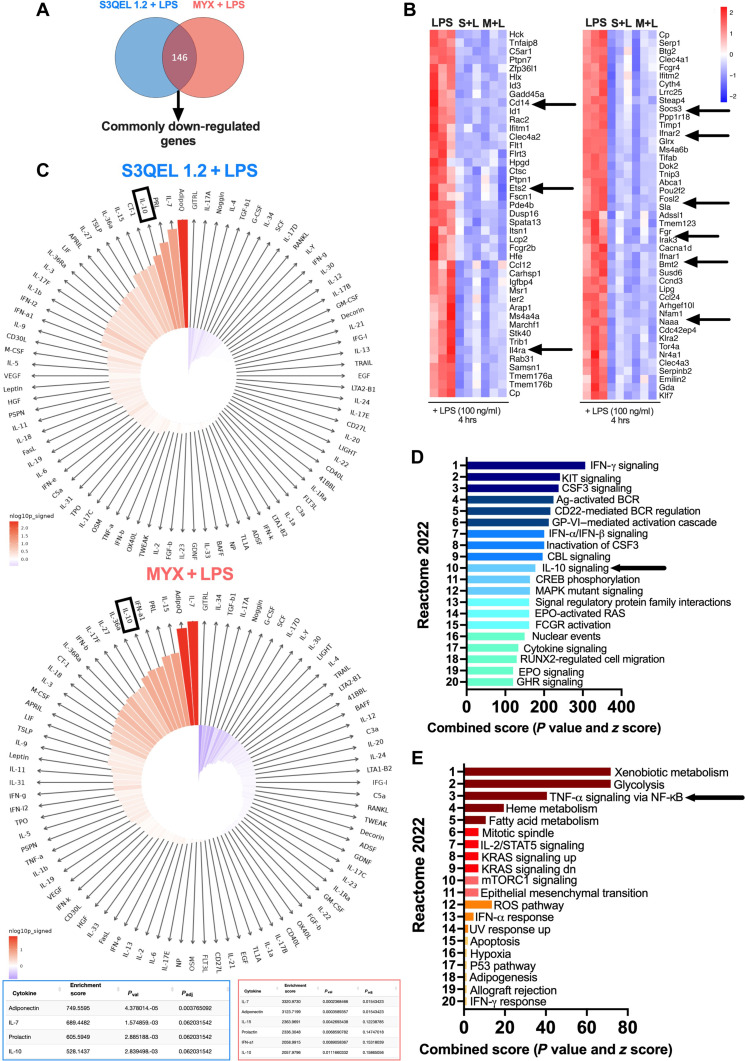
Complex III inhibition reveals a specific transcriptional signature in activated macrophages. (**A** to **E**) RNA sequencing of LPS-primed mouse macrophages (BMDMs) and pretreated (3 hours) with DMSO, S3QEL 1.2 (10 μM), and MYX (500 nM). (A) Venn diagram of commonly differentially expressed genes (146) between S3QEL 1.2 + LPS versus LPS and MYX + LPS versus LPS [adjusted *P* (*P*_adj_) < 0.1]. (B) Heatmap of the top 100 commonly up-regulated genes by LPS alone (red column) and down-regulated by both MYX and S3QEL 1.2 after LPS priming (second and third columns; in blue). hrs, hours. (C) IREA of differentially down-regulated genes by S3QEL 1.2 (top) and MYX (down) after LPS stimulation [adjusted *P* (P_adj_) < 0.05]. (D) and (E) GSEA of differentially regulated genes. (D) Overrepresentation analysis of RNA-seq data of BMDMs pretreated with S3QEL 1.2 or MYX (*n* = 3 from one independent experiment; LPS, 4 hours). Reactome pathway enrichment analysis of differentially down-regulated genes. (E) Overrepresentation analysis of RNA-seq data of BMDMs pretreated with S3QEL 1.2 or MYX (*n* = 3 from one independent experiment; LPS, 4 hours). Reactome pathway enrichment analysis of differentially up-regulated genes.

We then performed RNA sequencing (RNA-seq) analysis on BMDMs treated with S3QEL 1.2 and MYX and activated with LPS to investigate the contribution of complex III to the transcriptomic profile of macrophages. Differential analysis identified 146 genes, which were commonly impaired by both compounds in LPS-treated macrophages (Fig. 2A). Particularly, genes coding for protein that are essential for inflammatory macrophage function, such as cluster of differentiation 14 (*CD14*), the transcription factors *Ets2* and *Fosl2*, the receptors for interferon-α/β *IFNAR1* and *IFNAR2*, the ROS-activated Fgr kinase, NFAT-activating protein with ITAM motif 1 (*Nfam1*), *IL4r*, and suppressor of cytokine signaling 3 (*Socs3*), showed lowered expression, as indicated by arrows (Fig. 2B). Inhibition of complex III by MYX and S3QEL 1.2 boosted certain genes, including that encoding growth differentiation factor 15 (*GDF15*) (fig. S2, A and B). We focused our attention on the cytokine profile of activated macrophages and performed immune-responsive enrichment analysis (IREA) using our RNA-seq dataset. IREA is an approach based on a dictionary of single-cell transcriptomic profiles of 17 immune cell types in response to 86 different cytokines in vivo (publicly available data) (*18*). The analysis revealed that *IL-10* was among the cytokines whose down-regulation by S3QEL 1.2 and MYX after LPS stimulation was prominent (Fig. 2C). In addition, gene set enrichment analysis (GSEA) revealed that genes involved in IL-10 receptor signaling target genes were among the 20 most down-regulated terms (Fig. 1D) and genes contributing to tumor necrosis factor–α (TNF-α) and nuclear factor κB (NF-κB) signaling were among the 20 most up-regulated terms (Fig. 1E). IL-10 is well known to limit TNF-α production (*19*), hence this regulation found in our results.

### The inhibition of complex III decreases IL-10 production in activated macrophages

**Fig. 3.**
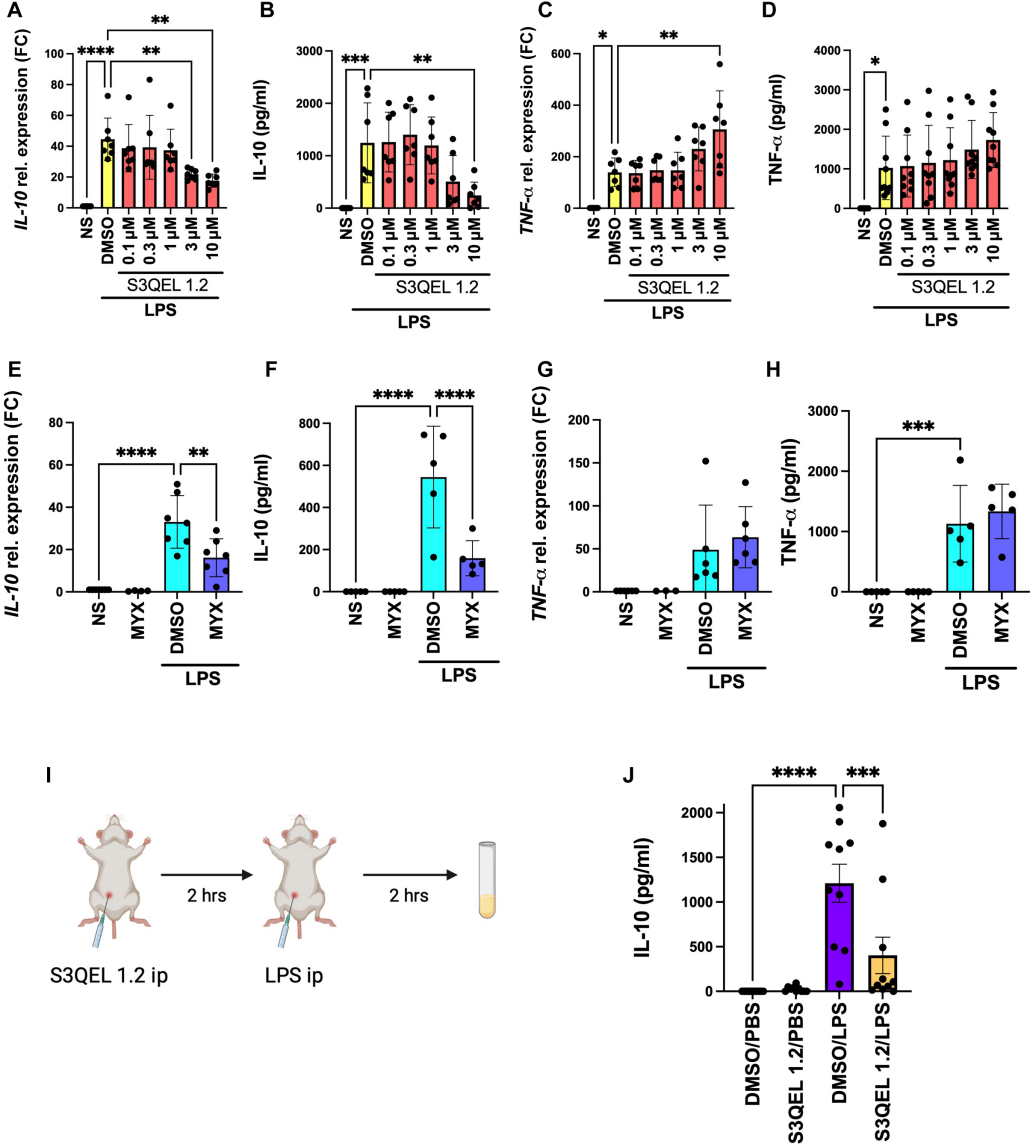
The inhibition of complex III decreases LPS-induced IL-10 expression in macrophages. (**A** to **D**) BMDMs were pretreated with DMSO or S3QEL 1.2 (0.1 to 10 μM) for 3 hours before LPS (100 ng/ml) stimulation for 4 hours, and cell lysates and supernatants were harvested. (A) and (B) Quantification of *IL-10* mRNA (A) and released protein (B) by RT-qPCR, relative to the *Rps18* housekeeping gene, and ELISA. (C) and (D) Quantification of *TNF-*α mRNA (C) and released protein (D) by RT-qPCR, relative to the *Rps18* housekeeping gene, and ELISA. FC, fold change. (**E** to **H**) BMDMs were pretreated with DMSO and MYX (500 nM) for 3 hours before LPS (100 ng/ml) stimulation for 4 hours, and cell lysates and supernatants were harvested. (E) and (F) Quantification of *IL-10* mRNA (E) and released protein (F) by RT-qPCR, relative to the *Rps18* housekeeping gene, and ELISA. (G) and (H) Quantification of *TNF-*α mRNA (G) and released protein (H) by RT-qPCR, relative to the *Rps18* housekeeping gene, and ELISA. (**I**) Schematic diagram of in vivo procedure and sample collection. Mice were intraperitoneally (ip) injected with PBS or S3QEL 1.2 (1 mg/kg) for 2 hours, followed by LPS (2.5 mg/kg) for 2 hours. Blood (then serum) was collected. hrs, hours. (**J**) Quantification of IL-10 by ELISA in the serum. Data from (A) to (H) are expressed as means ± SEM for *n* = 5 to 9 from three independent experiments. Data from (J) are expressed as means ± SEM (*n* = 10 per group within two independent in vivo experiments). *P* values were calculated using one-way ANOVA for multiple comparisons. Differences were considered statistically significant at *P < 0.05, **P < 0.01, ***P < 0.001, and ****P < 0.0001.

Because of the prominence of IL-10 in our analysis and the reported role of ROS in its regulation, we focused our attention on IL-10. We analyzed *IL-10* transcripts by reverse transcription quantitative polymerase chain reaction (RT-qPCR) and released form by enzyme-linked immunosorbent assay (ELISA), and we found that S3QEL 1.2 decreased LPS-induced IL-10 production in a dose-dependent manner (Fig. 3, A and B) and increased TNF-α (Fig. 3, C and D), as we had observed in the RNA-seq analysis. MYX had the same effects on *IL-10* mRNA and protein expression (Fig. 3, E and F) but curiously did not increase TNF-α (Fig. 3, G and H). We confirmed the redox-sensitive regulation of IL-10 with the pretreatment of macrophages with the mitochondria-targeted antioxidants MitoTEMPO and MitoQ, which, in the case of MitoQ, lowered IL-10 expression in response to LPS (fig. S3, A to D). We also tested the glutathione precursor *N*-acetyl cysteine (NAC), which impaired LPS-induced IL-10 expression especially at 1, 5, and 10 mM (fig. S3, E and F). Moreover, IL-10 regulation appeared to be complex I independent because pretreatment of activated BMDMs with rotenone, an inhibitor of complex I, and S1QEL 1.1, which impairs ROS production from complex I (*20*), did not substantially decrease *IL-10* mRNA or protein production (fig. S3, A, B, G, and H).

We next tested S3QEL 1.2 in vivo. S3QEL 1.2 has been used in a few studies where it was administered to mice in drinking water (*21*). Here, mice were injected intraperitoneally with S3QEL 1.2 and then injected with LPS after 2 hours (Fig. 3I). LPS-induced IL-10 in the serum was inhibited in the S3QEL 1.2–treated animals (Fig. 3J). Also, TNF-α was increased in serum after LPS injection, but S3QEL 1.2 did not have an inhibitory effect on it (fig. S4A).

We also analyzed S3QEL 1.2 effects on IL-10 produced by T_H_1 lymphocytes. S3QEL 1.2 decreased IL-10 release after T_H_1 cell polarization (fig. S5, A and B). Furthermore, MYX also suppressed *IL10* expression and release in LPS-stimulated macrophages derived from human peripheral blood mononuclear cells (PBMCs) (fig. S6, A and C). S3QEL 1.2 showed a trend toward a decrease in IL-10 at 0.1 and 0.5 μM (fig. S6, B and D). These results indicate that complex III–regulated IL-10 production is also evident in human cells.

### IL-10 suppression depends on inhibition of the AP1 subunit Fos

**Fig. 4.**
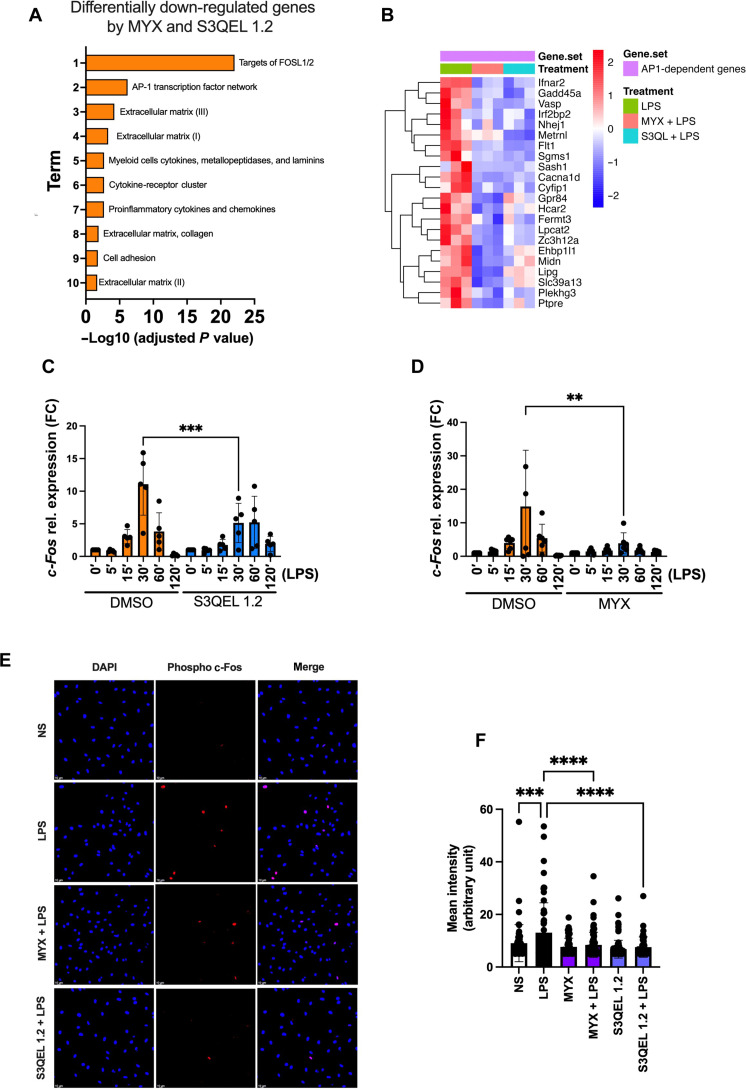
Complex III–dependent IL-10 regulation is mediated by the c-Fos subunit of the AP1 transcription factor. (**A** and **B**) Overrepresentation analysis of RNA-seq data of BMDMs pretreated with SQ3EL 1.2 or MYX (data are from *n* = 3 from one independent experiment; LPS, 4 hours). (A) EnrichR transcription factor enrichment analysis of differentially down-regulated genes. (B) Heatmap of significantly down-regulated AP1 target genes with adjusted *P* (P_adj_) < 0.05. (**C** and **D**) BMDMs were pretreated with DMSO, MYX (500 nM), or S3QEL 1.2 (10 μM) for 1 hour before LPS (100 ng/ml) stimulation for 0 to 120 min, and cell lysates were harvested. Quantification of c-*Fos* mRNA by RT-qPCR, relative to the *Rps18* housekeeping gene, in BMDMs pretreated with S3QEL 1.2 (C) or MYX (D) and stimulated with LPS. (**E** and **F**) Confocal microscopy images (E) and quantification (F) of BMDMs pretreated with DMSO, MYX (500 nM), or S3QEL 1.2 (10 μM) for 1 hour before LPS (100 ng/ml) stimulation for 2 hours. After that, cells were fixed and stained for phospho-c-Fos to assess cellular localization. Merge depicts phospho-c-Fos (red) together with nuclear staining (DAPI). Data in (C) and (D) are means ± SEM for *n* = 5 from three independent experiments. Data in (F) are means ± SD from one of the experiments in (E), showing the activated cells giving a fluorescence signal. Confocal images are representative of two independent experiments. *P* values were calculated using one-way ANOVA for multiple comparisons. Differences were considered statistically significant at **P < 0.01, ***P < 0.001, and ****P < 0.0001.

From the RNA-seq dataset previously analyzed, we performed transcription factor enrichment analysis, which revealed that the expression of *Fosl1* and *Fosl2*, canonical subunits of the transcription factor AP1, was inhibited by S3QEL 1.2 and MYX (Fig. 4A). Furthermore, we found that many of the genes that were down-regulated by S3QEL 1.2 and MYX were Fos-dependent target genes (Fig. 4B). We next analyzed the transcriptional levels of *c-Fos* by RT-qPCR, and we found that S3QEL 1.2 and MYX also diminished *c-Fos* levels after 30 min of LPS stimulation (Fig. 4, C and D). We also analyzed c-Jun, which is another subunit of AP1 and works with c-Fos to form a dimer, moves to the nucleus, and binds to the DNA. *c-Jun* mRNA expression after 30 min of LPS stimulation was decreased upon S3QEL 1.2 (fig. S7A) and MYX (fig. S7B) pretreatment. Subsequent confocal microscopy, performed to assess c-Fos activation and subsequent nuclear translocation, confirmed that the pretreatment with S3QEL 1.2 and MYX diminished the LPS-induced presence of phospho-c-Fos in the nucleus (Fig. 4, E and F). Together, these results suggest that the imperfect electron transport–derived ROS perturb AP1, which could likely block IL-10 production by LPS-activated macrophages. Since AP1 is being inhibited by S3QEL 1.2, and since *IL-10* is an AP1-dependent gene, this may be part of the mechanism of IL-10 repression, particularly as AP1 is known to be ROS sensitive (*22*).

### S3QEL 1.2 impairs tumor growth and elicit an immune cell response in mice in a B16F10 in vivo model

**Fig. 5.**
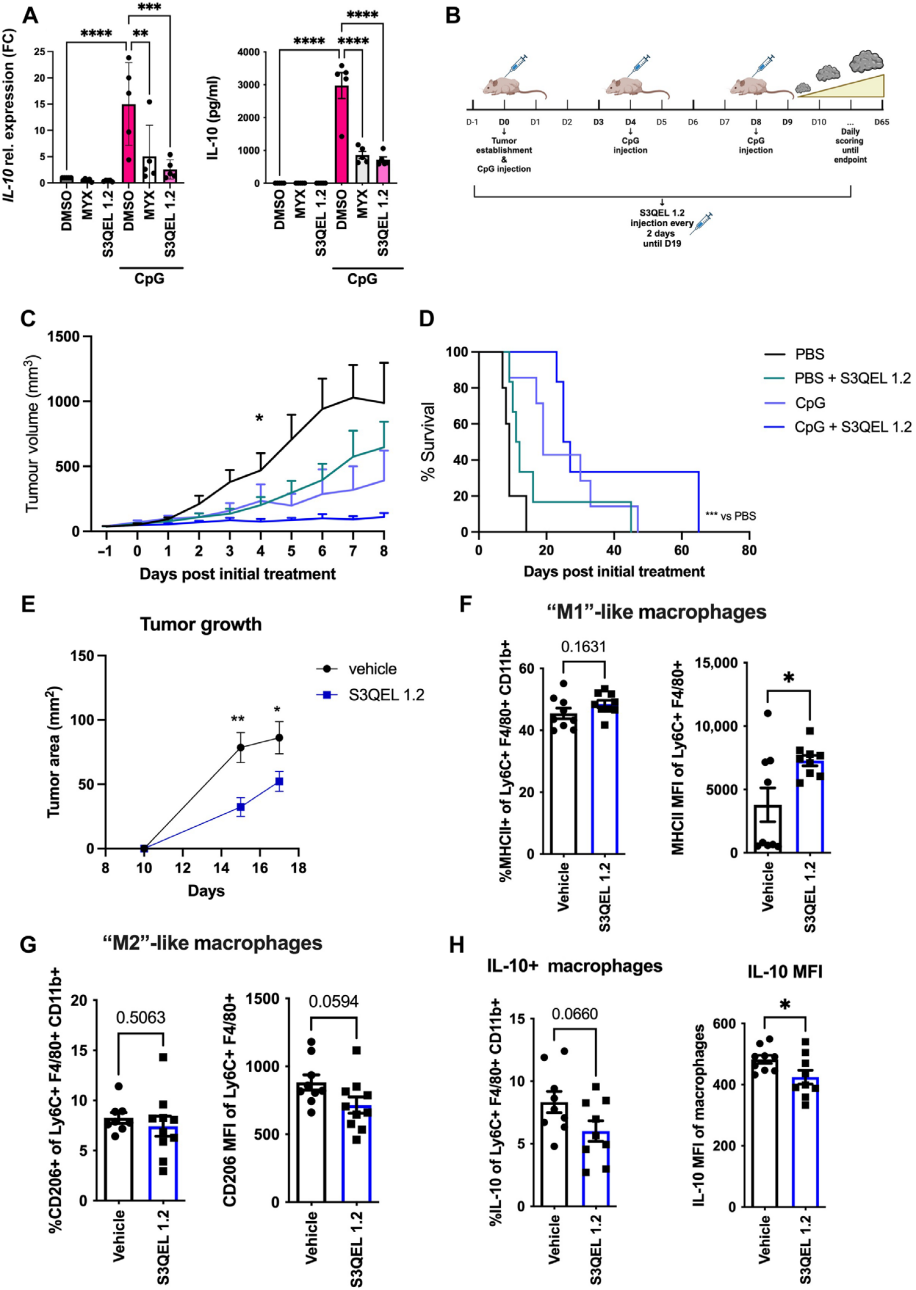
S3QEL 1.2 administration ameliorates survival and inhibits melanoma progression in mice in vivo. (**A**) BMDMs were pretreated with DMSO, MYX (500 nM), or S3QEL 1.2 (10 μM) for 3 hours, stimulated with CpG (1 μg/ml) for 4 hours, and cell lysates and supernatants were harvested. Quantification of *IL-10* mRNA by RT-qPCR, relative to the *Rps18* housekeeping gene, and of IL-10 levels by ELISA. (**B**) Schematic diagram of the melanoma in vivo model. Mice were challenged with 5 × 10^6^ B16F10 cells subcutaneously and administered with PBS, S3QEL 1.2 (1 mg/kg), and/or CpG (2.5 mg/kg). Tumors were measured daily. D, day. (**C**) Mean initial tumor growth curve relative to tumor volume from day −1 to day 8. (**D**) Kaplan-Meier survival graph up to day 65 (end of experiment). (**E**) Tumor growth rate curve relative to tumor area from day 0 to day 17. (**F** to **H**) Immune cells infiltrating the tumor were analyzed by flow cytometry. Cells expressing the surface markers Ly6C, F4/80, and CD11b showing the percentage of MHCII^+^ macrophages and mean fluorescence intensity (MFI) of MHCII (F), the percentage of CD206^+^ macrophages and MFI of CD206 (G), and the percentage of IL-10^+^ macrophages and MFI of IL-10 (H). Data from (A) are means ± SEM for *n* = 5 from three independent experiments. Data from (C) and (D) are means ± SEM for *n* = 5 for the PBS group, *n* = 6 for the S3QEL 1.2 group, *n* = 7 for the CpG group, and *n* = 6 for the S3QEL 1.2 + CpG group. Statistical significance for survival analysis was determined by Mantel-Cox test. Statistical significance for tumor growth analysis was determined by two-way ANOVA; asterisks are for *P* < 0.05 in the group’s comparison. Data from (E) to (H) are means ± SD for *n* = 9 from one independent in vivo experiment. *P* values were calculated using two-tailed unpaired Student’s *t* test. Differences were statistically significant at *P < 0.05, **P < 0.01, ***P < 0.001, and ****P < 0.0001.

IL-10 has been shown to be immunosuppressive in several tumor models and promoting tumor survival, notably in the B16F10 melanoma model (*23*). We therefore tested S3QEL 1.2 in this model alone (by intraperitoneal injection) or in combination with the immunostimulatory agent CpG (*24*). We first confirmed that similar to LPS, S3QEL 1.2 would inhibit IL-10 induction by CpG DNA in BMDMs, as shown in Fig. 5A. MYX was also inhibitory (Fig. 5A). The B16F10 model involved treating tumor-bearing mice with CpG DNA intratumorally and repeated intraperitoneal injections of S3QEL 1.2 (Fig. 5B). As shown in Fig. 5C, mice treated with S3QEL 1.2 or CpG alone limited tumor growth but had the most marked effect when given in combination. Similarly, as shown in Fig. 5D, both agents promoted survival with the combination, resulting in the longest survival time. Last, we analyzed by flow cytometry the immune cell subsets infiltrating the tumor [the gating strategy for myeloid cell and non–myeloid cell subsets is shown in fig. S8 (A to D)]. Tumor burden was decreased in the S3QEL 1.2 alone–treated group (Fig. 5E). Cell counts and tumor mass were also lowered (fig. S9, A to C). M1-like macrophages infiltrating the tumor showed increased expression of major histocompatibility complex class II (MHCII) molecules (Fig. 5F), relevant for the presentation of tumor antigens to CD4^+^ T cells, after S3QEL 1.2 administration while M2-like macrophages presence showed a moderate decrease in the same group (Fig. 5G). IL-10 production by tumor-infiltrating macrophages was impaired by S3QEL 1.2 (Fig. 5H). The number of tumor-infiltrated leukocytes was increased after S3QEL 1.2 administration (fig. S9D), while the number of myeloid cells, neutrophils, and monocyte-macrophages remained the same (fig. S9, E to G). The amounts of dendritic cells were lower in the S3QEL 1.2–treated group (fig. S9H). This last subset of cells did not show any augmented expression of MHCII molecules in mice treated with S3QEL 1.2 (fig. S9I). Last, we also investigated the effect of S3QEL 1.2 on B16F10 melanoma cells in vitro. The highest concentration of S3QEL 1.2 impaired the proliferation of B16F10 cells but did not block OXPHOS and complex III–specific activity, in contrast to MYX, which impaired all these processes (fig. S10, A to F), as assessed by metabolic flux analysis and crystal violet assay. The effect of S3QEL 1.2 on tumor growth may therefore be a combination of a dual effect on tumor cell proliferation and inhibition of IL-10 to promote antitumor immunity.

## DISCUSSION

Mitochondrial complex III and its involvement in regulating immune function have only recently begun to be examined. Mice lacking the Rieske iron sulfur protein subunit of complex III showed the diminished suppressive capacity of T_regs_, together with decreased expression of markers of T_regs_ such as EOS and CTLA-4, without changing their Foxp3 and HELIOS expression (*11*). In another study, complex III knockout mice had lower numbers of CD4^+^ and CD8^+^ T cells in the periphery, with less IL-2 expression (*12*).

Here, we have deployed S3QEL 1.2, a highly selective suppressor of complex III–derived ROS, which has been developed by Brand and colleagues (*15*). S3QELs were identified through an Amplex UltraRed–based detection system used to screen 635,000 small molecules against H_2_O_2_ production caused by electron leak at sites III_Qo_, I_Q_, and II_F_ in isolated muscle mitochondria. After the elimination of compounds that were unselective for site III_Qo_ or that inhibited energy metabolism, S3QELs were confirmed to potently and selectively suppress site III_Qo_ superoxide production without impairing any bioenergetic function, including mitochondrial membrane potential (ΔΨ_m_) (*15*). S3QEL 1.2 has been shown to prevent the accumulation of ROS produced by complex III and IL-6 production from alveolar macrophages exposed to particulate matter (*25*). In addition, in another study involving BMDMs activated by LPS and extracellular ATP, S3QEL 1.2 did not affect IL-1β production, suggesting a mechanism for NLRP3 inflammasome activation that is independent of complex III–derived ROS (*26*). However, a role for complex III–derived ROS in the production of other key cytokines from macrophages was not investigated. IL-10 appeared to be strongly affected by complex III inhibition in our data, and increasing literature is highlighting its dependency on mitochondrial ETC, although precise details are lacking (*27*–*29*).

Our findings show that complex III sustains IL-10 generation through intact respiration and production of ROS. Inhibition of complex II has been shown to block IL-10 production (*27*), possibly because of electron flow to complex III, preventing it from generating ROS. In addition, our bioinformatics analysis suggests that complex III can regulate the cellular antioxidant response and drive cytokine production via a plethora of pathways. From our findings, it is clear that complex III impairment up-regulates glycolysis as a possible compensatory mechanism to fuel ATP production in the absence of O_2_-dependent respiration.

The mechanism that we propose for the decrease in IL-10 expression by complex III impairment is down-regulation of the transcription factor AP1 and, especially, its subunit c-Fos (fig. S11). We hypothesize that ROS produced by complex III would oxidize specific cysteine residues on AP1 or its upstream regulators and subsequently activate them. The redox-sensitive nature of c-Fos has already been described by Wang and colleagues, who showed that the mitochondria-targeted antioxidant mitoTEMPO decreased the oxLDL-mediated abundance of c-Fos and phosphorylated c-Fos in smooth muscle cells (*30*). In another study, mitoTEMPO abolished the binding of AP1 to DNA in endothelial cells activated by lysophosphatidylcholine (*31*). As *c-Fos* and *c-Jun* transcripts are lowered by S3QEL 1.2 and MYX, we do not exclude the possibility of the regulation of proteins that control the stability of mRNAs here (*32*). However, further investigation is necessary to understand the exact mechanism for c-Fos regulation by ROS. Peroxiredoxins, which are antioxidant enzymes responsible for the maintenance of redox balance within the cell, and particularly peroxiredoxin-6, have been detected as interactors of AP1 in mice (*33*).

IL-10 overexpression is a key feature of aggressive and highly proliferative tumors, such as melanoma and colorectal cancer (*34*). Because tumor-associated macrophages (TAMs), together with myeloid-derived suppressor cells (MDSCs) and T_regs_ and B cells, are the main sources of IL-10 in the tumor microenvironment and contribute to the abolishment of the cytotoxic T cell response (*35*), we hypothesized that S3QEL 1.2 could have a beneficial effect through the dampening of tumor-driven immunotolerance mediated by IL-10. We therefore tested S3QEL 1.2 in the B16F10 melanoma in vivo model, where neutralization of the IL-10 receptor with an antibody has been shown to boost antitumor immunity, especially in combination with CpG DNA (*36*). The beneficial effect of S3QEL 1.2 here is likely to be via inhibition of IL-10 production by macrophages. However, other pro-inflammatory cytokines and chemokines involved in the cross-talk between tumor and immune cells are differentially regulated after complex III impairment. For example, *CXCL9* is up-regulated by S3QEL 1.2 pretreatment (fig. S2B) and it has been shown to define macrophage polarization, thus an antitumor immune response, in human colorectal cancers (*37*). In addition, *IL-7* and *IL-15* are other two cytokines that are down-regulated after complex III impairment (Fig. 2C). Previous findings have described IL-7 as fundamental for the activation of myeloid cell populations in mice bearing CT26 tumors (*38*) and for tumor invasiveness in prostate cancer (*39*), while IL-15–dependent signaling in tumor-associated macrophages inhibited CD8^+^ T cell recruitment in breast cancer (*40*). Taking into consideration the complexity of the tumor microenvironment, we cannot rule out possible effects of S3QEL 1.2 on other cell types and we believe that S3QEL 1.2 might also inhibit proliferation of the B16F10 melanoma cells themselves. Therefore, the treatment of macrophages before in vivo injection with tumor cells, as previously deployed (*41*), or the use of IL-10 conditional knockout models may represent other options required to further elucidate the specific mechanism of action and target of S3QEL 1.2.

In conclusion, our work provides evidence for complex III–derived ROS in regulating IL-10 production in macrophages, broadening the existing knowledge about ETC-mediated rewiring of myeloid cells (*42*). Moreover, S3QEL 1.2 could be a useful tool for dissecting immunotolerance in the context of tumor development and progression. Recently, a complex I inhibitor was tested in acute myeloid leukemia and advanced solid tumors but proved to be neurotoxic (*43*). The advantage of S3QEL 1.2 is its limited effect on respiration, and therefore, it may be less toxic in the treatment of cancer.

## MATERIALS AND METHODS

### Animal details

All mice used for the in vitro experiments were on a C57BL/6JOlaHsd background, unless stated below. Wild-type mice, when needed, were purchased from Envigo. In vivo experiments were performed with 8-week-old female mice bred under specific pathogen–free conditions, under the license and approval of the local animal research ethics committee (Health Products Regulatory Authority) under European Union regulations. The first two in vivo LPS models were performed with 6-to-12-week-old C57BL/6JOlaHsd mice, and littermates were randomly assigned to experimental groups. All animal procedures were ethically approved by the Trinity College Dublin Animal Research Ethics Committee before experimentation and conformed to the directive 2010/63/EU of the European Parliament (authorization AE19136/P148).

The first B16F10 melanoma in vivo model was performed with 6-to-12-week-old C57BL/6J mice, which were randomly assigned to experimental groups. All animal procedures were ethically approved by the Trinity College Dublin Animal Research Ethics Committee before experimentation and conformed to the directive 2010/63/EU of the European Parliament (authorization AE19136/P172).

The second B16F10 melanoma in vivo model was performed in the animal facilities of the Medical University of Graz. Wild-type C57BL/6J mice were purchased from Charles River Laboratories. Ethical approval for animal experimental protocols was granted by the Austrian Federal Ministry of Science and Research (protocol number: 2022-0.748.851/2024-0.783.500).

### B16F10 in vivo model

B16F10 cells (American Type Culture Collection) were maintained in Dulbecco’s modified Eagle’s medium (DMEM) (Gibco) containing 10% fetal bovine serum (FBS) and 1% penicillin-streptomycin at 37°C and 5% CO_2_ in a humified incubator. Mice were injected subcutaneously with 3.5 × 10^5^ B16F10 cells on the right flank, which has been previously shaved. Tumor growth was measured daily using a digital caliper, and the tumor volume was calculated as [Length × (Width)^2^]/2. Upon reaching 30 to 60 mm^3^, mice were randomly assigned to experimental treatment groups and received intratumoral injections of phosphate-buffered saline (PBS) or CpG ODN 1826 (2.5 mg/kg each treatment), with treatments repeated 4 days later on day 4 and on day 8 for a total of three intratumoral treatments. In one experiment, two groups of mice received S3QEL 1.2 (1 mg/kg; 5% in PBS) via the intraperitoneal route every 2 days from day one before the first intratumoral treatment until day 19. Mice were culled upon reaching the predetermined humane end point of tumor diameter >15 mm or, in the case of ulceration, with survival analyzed using Kaplan-Meier analysis.

In the second in vivo experiment, B16F10 cells (American Type Culture Collection) were maintained in DMEM (Gibco) containing 10% FBS and 1% penicillin-streptomycin at 37°C and 5% CO_2_ in a humified incubator. Mice were injected subcutaneously with 5 × 10^6^ B16F10 cells on the right flank, which has been previously shaved. Tumor growth was measured daily using a digital caliper, and the tumor volume was calculated as [Length × (Width)^2^]/2. Upon reaching 30 to 60 mm^3^, mice were randomly assigned to experimental treatment groups and received S3QEL 1.2 or PBS intraperitoneally. S3QEL 1.2 was dissolved in a vehicle containing dimethyl sulfoxide (DMSO):ethanol:Kolliphor EL (Sigma-Aldrich) at a 1:1:2 ratio and divided into aliquots; a vehicle control containing no drug was also prepared. Before injection, an aliquot of S3QEL 1.2 or vehicle in DMSO/ethanol/Kolliphor was added to pre-prepared 5% (w/v) glucose in water at a 1:6.66667 ratio. Two hundred microliters of this mixture per mouse was injected intraperitoneally every other day to give a final S3QEL 1.2 concentration of 1 mg/kg per mouse.

### Flow cytometry of tumor single-cell suspensions

B16F10 tumors were removed, mechanically minced using surgical scissors, and subsequently digested with deoxyribonuclease I (160 U/ml; Worthington) and collagenase (4.5 U/ml; Worthington) for 30 min at 37°C, with vortexing and shaking. Samples were then passed through a 40-μm cell strainer, washed in PBS and 2% FBS, and resuspended in flow staining buffer (PBS containing 2% FBS and 2 mM EDTA). Cells were counted on the EVE automated cell counter (NanoEntek).

A total of 3 × 10^6^ cells per sample in 100 μl of staining buffer were used for flow cytometry staining. Single-cell suspensions were incubated for 20 min in fixable viability dye eFluor 780 (eBioscience) according to the manufacturer’s instructions. Fc receptor blocking was then performed using TruStain FcX (BioLegend) according to the manufacturer’s instructions. For surface staining, cells were incubated for 30 min at 4°C in the dark in flow staining buffer with the following antibodies (all purchased from BD/BioLegend): CD45-AF700 (clone 30-F11, 560510), CD3-BV421 (clone 17A2, 564008), CD4-BUV496 (clone GK1.5, 612952), CD8-PE-Cy7 (clone 53-6.7, 561097), NKp46-BV510 (clone 29A1.4, 563455), CD19-BV711 (clone 1D3, 563157), CD11b-BUV737 (clone M1/70, 612801), Ly6C-APC (clone AL21, 560595), Ly6G-PE/Dazzle594 (clone 1A8, 127648), CD11c-BV605 (clone N418, 117333), F4/80-BUV395 (clone T45-2342, 565614), SiglecF-PE (clone E50-2440, 552126), CD206-FITC (clone C068C2, 141704), and MHCII-PerCP-Cy5.5 (clone M5/114.15.2, 107626). Single stains and fluorescence minus one (FMO) controls were also used. Cells were then washed twice in flow staining buffer, fixed using IC Fixation Buffer (eBioscience) for 10 min, washed again twice, and resuspended in flow staining buffer.

For intracellular staining of IL-10, 3 × 10^6^ cells of each single-cell suspension sample were seeded into a 96-well U-bottom plate with RPMI containing GolgiStop (1.5 μl/ml, BD Biosciences) and incubated for 1 hour in a 37°C cell culture incubator. Fixable viability dye staining, Fc receptor blocking, and surface antibody staining were performed as above. Cells were then permeabilized and fixed using Perm/Fix Buffer (BD Biosciences) for 30 min, followed by subsequent staining for IL-10 using an IL-10-PE antibody (BioLegend) for 30 min. Single stains and FMO controls were also used. Cells were then washed twice in Perm/Fix buffer (prepared according to the manufacturer’s instructions; BD Biosciences), and resuspended in flow staining buffer.

Samples were analyzed on a BD LSRFortessa flow cytometer using FACSDiva software (BD Biosciences). Analyses were performed using FlowJo version 10.10 software, and FMO and single stains were used to compensate and define gates.

### Flow cytometry of T_H_1 lymphocytes

After activation, the cells were then washed and stained for 10 min at room temperature with fixable viability dye (Thermo Fisher Scientific) for dead cell exclusion and fluorochrome-labeled monoclonal antibodies against surface cell markers (CD4-PerCPCy5.5, clone RM4-4, 116011). Afterward, cells were washed and fixed for 15 min at room temperature using the eBioscience Foxp3 Fixation/Permeabilization Kit. After other washing steps, cells were permeabilized using the same kit and stained intracellularly with monoclonal antibodies (IFN-γ-APC, clone XMG1.2, 505810; and IL-10-APCCy7, JES5-16E3, 505035). Data were acquired on FACSCanto II machines (BD Biosciences) and analyzed using FlowJo software (Tree Star), and unstained single-stained controls were used to compensate and define gates.

### Generation of murine BMDMs

Mice (6- to 12-week-old) were euthanized in a CO_2_ chamber, and death was confirmed by cervical dislocation [procedures previously described in (*44*, *45*)]. Bone marrow was subsequently harvested from the tibia, femur, and ilium. For monocyte/macrophage isolation, cells were differentiated in DMEM containing L929 supernatant (20%), FBS (10%), and penicillin-streptomycin (1%) for 6 days, after which cells were counted and plated at 0.5 × 10^6^ cells/ml in 10% L929 supernatant, unless otherwise stated.

### Isolation of human PBMCs

Human blood samples from healthy donors were collected and processed at the School of Biochemistry and Immunology at the Trinity Biomedical Sciences Institute (TCD). Blood samples were obtained anonymously, and written informed consent for the use of blood for research purposes was obtained from the donors. All the procedures involving experiments on human samples were approved by the School of Biochemistry and Immunology Research Ethics Committee (TCD). Experiments were conducted according to the TCD guide on good research practice, which follows the guidelines detailed in the National Institutes of Health Belmont Report (1978) and the Declaration of Helsinki. Whole blood (30 ml) was layered on 20 ml of Lymphoprep (Axis-Shield), followed by centrifugation for 20 min at 400*g* with the brake off, after which the upper plasma layer was removed and discarded. The layer of mononuclear cells at the plasma-density gradient medium interface was retained, and 20 ml of PBS was added. Cells were centrifuged for 8 min at 300*g*, and the resulting supernatant was removed and discarded. The remaining pellet of mononuclear cells was resuspended, counted, and plated at 1 × 10^6^ cells/ml in RPMI supplemented with FBS (10%) and penicillin-streptomycin (1%).

### Generation of human macrophages

PBMCs were obtained, and CD14^+^ monocytes were isolated using a MagniSort Human CD14 Positive Selection kit (Thermo Fisher Scientific) according to the manufacturer’s protocol. CD14 monocytes were then differentiated in T-175 flasks in RPMI containing FBS (10%), penicillin-streptomycin (1%), and recombinant human macrophage colony-stimulating factor (1:10,000). After 6 days, the supernatant was discarded, cells were scraped and counted, and human monocyte-derived macrophages were plated in 12-well plates at 1 × 10^6^ cells/ml in RPMI containing FBS (10%) and penicillin-streptomycin (1%).

### In vitro T_H_1 cell differentiation

Naive CD4^+^CD25^−^ T cells were purified from spleens of wild-type C57BL/6OlaHsd mice with the naïve CD4 T cell isolation kit (Miltenyi Biotec) by using an AutoMACS magnetic cell sorter (Miltenyi Biotec) according to the manufacturer’s protocol. Purified cells were activated with anti-CD3ε:CD28 (3 and 2 μg/ml, respectively; BD Biosciences) coated on an F-bottomed plate (10^5^ per well). Skewing conditions were rmIL-12 and rmIL-2 (both 20 ng/ml; R&D Systems) for T_H_1 cells for 72 hours. When indicated, MYX (50, 500, or 5000 nM) and S3QEL 1.2 (0.1, 1, or 10 μM) were added to the polarization media. Subsequently, T_H_1 cells were activated by phorbol 12-myristate 13-acetate/ionomycin stimulation (50 and 500 ng/ml, respectively) in the presence of StopGolgi solution (1.5 μl/ml, BD Biosciences) at 37°C in a humidified 5% CO_2_ chamber for 3 hours.

### Reagents

MYX was dissolved in DMSO, both purchased from Sigma-Aldrich. S3QEL 1.2 and S1QEL 1.1 were provided by M. D. Brand (Buck Institute for Research on Aging, Novato, CA, US). LPS from *Escherichia coli* (ALX-581-010-L002) was purchased from Enzo Life Sciences for in vitro experiments. NAC and MitoTEMPO were purchased by Sigma-Aldrich. CpG ODN 1826 (130-109-373/374) was purchased from Miltenyi Biotec for in vitro and in vivo experiments. LPS from *E. coli* (L4524) was purchased from Sigma-Aldrich for in vivo experiments. MitoTEMPO was purchased from Sigma-Aldrich (0737), and MitoQ was purchased from Abcam (ab285406) for in vitro expperiments. Rotenone (Sigma-Aldrich, 8875) and piericidin A (MedChemExpress, HY-114936) were also used for in vitro experiments. NAC was purchased from Merck (A7250) for in vitro experiments.

### RNA isolation and real-time qPCR

Total RNA was isolated using the PureLink RNA Mini Kit (Invitrogen) and quantified using a Nanodrop 2000 UV-visible spectrophotometer. cDNA was prepared using total RNA (20 to 100 ng/μl) by RT-PCR using a high-capacity cDNA reverse transcription kit (Applied Biosystems) according to the manufacturer’s instructions. Real-time qPCR was performed with an ABI 7500 Fast real-time PCR system (Applied Biosystems) on cDNA using SYBR Green (Invitrogen). Data were normalized to murine RPS18 as endogenous controls, and the mRNA expression fold change relative to controls was calculated using the 2^−ΔΔ*C*t^ method. All fold changes are expressed normalized to the untreated control. The following murine primers were used: *IL-1*β, 5′-GAGGACATGAGCACCTTCTTT-3′, 3′-GCCTGTAGTGCAGTTGTCTAA-5′; *TNF-*α, 5′-GCCTCTTCT-CATTCCTGCTT-3′, 3′-TGGGAACTTCTCATCCCTTTG-5′; *IL-10*, 5′-AGGCGCTGTCATCGATTT-3′, 3′-CACCTTGGTCTTGGAGCTTAT-5′; *IL-6*, 5′-CCACAGTCCTTCAGAGAGATACA-3′, 3′-CCTTCTGTGACTCCAGCTTATC-5′; *c-Fos*, 5′-GGGAATGGTGAAGACCGTGTCA-3′, 3′-GCAGCCATCTTATTCCGTTCCC-5′; *c-Jun*, 5′-CAGTCCAGCAATGGGCACATCA-3′, 3′-GGAAGCGTGTTCTGGCTATGCA-5′; *Act*β, 5′-GCCTTCCTTCTTGGGTATGG-3′, 3′-A GCACTGTGTTGGCATAGAG-5′; *RPS18*, 5′-GCGAGTACTCAACACCAACA-3′, 3′-CCTCAACACCACATGAGCATA-5′. The following human primers were used: *IL-10*, 5′-GTCCTTGCTGGAGGACTTTA-3′, 3′-ATGTCTGGGTCTTGGTTCTC-5′; *RPS13*, 5′-CGAAAGCATCTTGAGAGGAACA-3′, 3′-TCGAGCCAAACGGTGAATC-5′.

### RNA sequencing

BMDMs (three independent mice) were treated as indicated, and RNA was extracted as previously detailed. mRNA was extracted from total RNA using poly-T-oligo–attached magnetic beads. After fragmentation, the first-strand cDNA was synthesized using random hexamer primers, followed by second-strand cDNA synthesis using either deoxyuridine triphosphate for directional library or deoxythymidine triphosphate for nondirectional library. The library was checked with Qubit and real-time PCR for quantification and a bioanalyzer for size distribution detection. Quantified libraries were pooled and sequenced on the NovaSeq 6000 S4 (Illumina).

Raw data (raw reads) in fastq format were first processed through in-house (Novogene) perl scripts. In this step, clean data (clean reads) were obtained by removing reads containing an adapter, reads containing ploy-N, and low-quality reads from raw data. At the same time, Q20, Q30, and GC content were calculated. All the downstream analyses were based on the clean data with high quality. The index of the reference genome was built usingHisat2 version 2.0.5, and paired-end clean reads were aligned to the reference genome using Hisat2 version 2.0.5.

FeatureCounts version 1.5.0-p3 was used to count the read numbers mapped to each gene. Then, fragments per kilobase of transcript per million mapped reads of each gene were calculated on the basis of the length of the gene and read counts mapped to this gene.

Differential expression was analyzed using the DESeq2R package (1.20.0). The resulting *P* values were adjusted using Benjamini and Hochberg’s approach for controlling the false discovery rate. Genes with an adjusted *P* ≤ 0.05 found by DESeq2 were assigned as differentially expressed. All the analyses and heatmaps were performed using custom scripts in R version 4.3.1. GSEA was performed using the online EnrichR tool.

### ELISA

A DuoSet ELISA kit for IL-10 and TNF-α (R&D Systems) in the supernatant of BMDMs was purchased and used according to the manufacturer’s instructions with appropriately diluted cell supernatants added to each plate in duplicate or triplicate. For in vivo samples, a Quantikine ELISA kit for mouse IL-10 (R&D Systems) was used according to the manufacturer’s instructions with appropriately diluted serum added to each plate. Absorbance was read at 450 nm and quantified using a FLUOstar Optima plate reader. Corrected absorbance values were calculated by subtracting the background absorbance, and cytokine concentrations were subsequently obtained by extrapolation from a standard curve plotted on GraphPad Prism 10.1.1.

### MTT assay

BMDMs at a concentration of 1 × 10^5^ cells were seeded in each well of a 96-well F-bottom plate in duplicates or triplicates and treated accordingly. MTT was purchased from Sigma-Aldrich and reconstituted in PBS to a concentration of 1 mg/ml. On each plate, three wells had no cells but the medium only to serve as a blank. At the end of the treatment, cells were washed with 100 μl per well and 100 μl of MTT solution was added in each well. The plate was incubated for 2 hours at 37°C wrapped in tin foil. At the end of the incubation, the MTT solution was discarded, and the crystals were dissolved in 100 μl of DMSO solution in each well. The plate was incubated for 20 min at 37°C in the dark and read on a spectrophotometer at 595 nm of wavelength. Data were normalized to untreated and unstimulated cells.

### Extracellular flux analysis

A Seahorse XFe-96 extracellular flux analyzer (Seahorse Bioscience, Agilent Technologies) was used to define oxygen consumption rates (OCRs) and extracellular acidification rates. For monitoring OXPHOS, cells were plated at 1 × 10^5^ cells per well in XF DMEM (pH 7.4) supplemented with pyruvate, glutamine, and glucose. Mitochondrial perturbation experiments were carried out by sequential addition of oligomycin (oligo), carbonyl cyanide 4-(trifluoromethoxy) phenylhydrazone (FCCP), antimycin A (AA), and rotenone according to the manufacturer’s instructions. For monitoring glycolysis, cells were plated in XF DMEM (pH 7.4) supplemented with glutamine. Glycolysis perturbation experiments were carried out by sequential addition of glucose, oligo, and 2-deoxyglucose according to the manufacturer’s instructions. For monitoring the OCR of only intact mitochondria, cells were plated in MAS buffer (70 mM sucrose, 220 mM mannitol, 10 mM KH_2_PO_4_, 5 mM MgCl_2_, 2 mM Hepes, and 1 mM EGTA) and then treated with the XF plasma membrane permeabilizer (Seahorse, Agilent Technologies), followed by addition of 0.5 mM duroquinol, 1 mM adenosine 5′-diphosphate (ADP), 15 μM oligo, and 0.02 mM AA for monitoring complex III–driven respiration. In this case, the assay includes a cycle of mixing for 2′, waiting for 2′, and measuring for 3′ (repeated three times); duroquinol and ADP injections, followed by the same cycle repeated two times; then oligomycin injection, followed by the same cycle repeated two times; and AA injection, followed by the same cycle repeated two times. Changes in OCR on substrate addition were calculated relative to the preinjection rate.

### Western blotting

The supernatant was removed from cells after stimulation, and lysates were harvested in 30 to 50 μl of lysis buffer [0.125 M Tris (pH 6.8), 10% glycerol, 0.02% SDS, and 5% dithiothreitol]. Lysates were subsequently heated to 95°C for 5 min to denature proteins. SDS–polyacrylamide gel electrophoresis was used to resolve proteins by molecular weight. Samples were boiled at 95°C for 5 min before loading into a 5% stacking gel. The percentage resolving gel (8 to 12%) depended on the molecular weight of the given protein. The Bio-Rad gel running system was used to resolve proteins, and the Bio-Rad wet transfer system was used for the electrophoretic transfer of proteins onto the polyvinylidene difluoride membrane. After transfer, the membrane was incubated in blocking solution [5% milk powder or 5% bovine serum albumin (BSA) in Tris-buffered saline with Tween 20 (TBST)] for 1 hour and subsequently incubated in a primary antibody (5% milk powder or 5% BSA in TBST) rolling overnight at 4°C. The membrane was incubated for 1 hour with a secondary antibody (diluted in 5% milk powder or 5% BSA in TBST) at room temperature. Before visualization, the membrane was immersed in WesternBright ECL Spray (Advansta). Proteins were visualized on a ChemiDoc MPTM Imaging System (Bio-Rad), and both chemiluminescent and white light images were taken.

Quantification of Western blot images was performed using Image Lab Software (Bio-Rad). The adjusted band volume was calculated for each band, and for each experimental condition, this was presented as the target protein/housekeeping protein.

The following antibodies were used: antimouse c-Fos (2250), phospho-c-Fos (5348), c-Jun (9165), phospho-c-Jun (3270), GAPDH (glyceraldehyde-3-phosphate dehydrogenase; 2118), and α-tubulin (2144); they were purchased from Cell Signaling Technology. Total OXPHOS rodent Western blot antibody cocktail was purchased from Abcam (ab110413). Antimouse β-actin (A5316) was purchased from Sigma-Aldrich.

### Immunofluorescence and confocal microscopy

Cells (2.5 × 10^5^) were plated on 12-mm coverslips in 24-well plates. Cells were treated as required and washed three times with warm PBS. Cells were subsequently fixed for 10 min with 2% paraformaldehyde in PBS at 37°C. Cells were washed three times with PBS and permeabilized for 1 hour in block solution [1% BSA, glycine (22.52 mg/ml), and 0.1% Tween 20 in PBS]. Anti-phospho-c-Fos antibody (Cell Signaling Technology, 5348) was diluted 1:800 in block solution and incubated with cells overnight at 4°C. Cells were washed three times with PBS for 5 min per wash. A mix containing Alexa 647–conjugated goat antirabbit IgG (H + L) antibody (1:1000) and DAPI (4′,6-diamidino-2-phenylindole; 1:1000, Thermo Fisher Scientific, 62248) was subsequently added to cells for 90 min at room temperature in the dark. Cells were subsequently washed three times with PBS for 5 min per wash. Coverslips were mounted onto microscope slides using 10 to 20 μl of ProLong Gold antifade reagent (Thermo Fisher Scientific, P36930). Slides were imaged using a Leica SP8 scanning confocal microscope with a ×40.0 objective. Images were analyzed using LAS X Life Science Microscope Software Platform (Leica). The same microscope instrument settings were used for all samples, and all images were analyzed using the same settings. Quantification of phospho-c-Fos signal intensity was performed using Imaris software 10.1.1. The mean signal intensity was calculated for individual cells in single-color images and displayed relative to the signal intensity of control cells.

### Amplex Red H_2_O_2_ measurement

BMDMs were seeded in a 96-well plate with black opaque inserts with a concentration of 1 × 10^5^ cells per well in duplicates. Cells were treated accordingly and stimulated with LPS for 48 hours (100 ng/ml) and with MitoTEMPO for 48 hours (5 mM). Cells were treated with S3QEL 1.2 (10 μM) and MYX (500νM) on the day of the assay, and media were changed to KRPG Buffer. The H_2_O_2_ standard was prepared as per the manufacturer’s instructions. Fifty microliters of 100 μM Amplex Red and HRP working solution (0.2 U/ml) were added to the wells of the H_2_O_2_ standard. One hundred microliters of 50 μM Amplex Red and HRP working solution (0.1 U/ml) were added to the wells containing the samples. Fluorescence with excitation between 450 and 490 nm and emission of 590 nm was read at the beginning of the reaction, after 30 min, and then every hour for maximum 3 hours. The fluorescence of the samples was extrapolated from the H_2_O_2_ standard curve and normalized to the untreated sample.

### LPS-induced model of inflammation

C57BL/6OlaHsd mice were pretreated intraperitoneally with S3QEL 1.2 or DMSO vehicle control at the final concentration of 1 mg/kg (1:10 in PBS) for 2 hours and then injected intraperitoneally with LPS or PBS vehicle control at the final concentration of 2.5 mg/kg for 2 hours. After 4 hours, mice were euthanized and blood was collected retro-orbitally. Peritoneal exudate cells were harvested in PBS. Blood was centrifuged at 4°C for 10 min at 5000 rcf, and then the supernatant (serum) was kept at −80°C for further analysis by ELISA. Peritoneal exudate cells were pelleted and lysed in RNA lysis buffer (Invitrogen) for further analysis by qPCR.

### Quantification and statistical analysis

Details of all statistical analyses performed can be found in the figure legends. Data were expressed as means ± SEM, unless stated otherwise. *P* values were calculated using two-tailed Student’s *t* test for pairwise comparison of variables and one-way analysis of variance (ANOVA) for multiple comparisons of variables. A Sidak’s multiple comparisons test was used as a post test when performing an ANOVA. A Mantel-Cox test was used for log-rank analysis of the survival Kaplan-Meier curve. A confidence interval of 95% was used for all statistical tests. For statistical significance, exact *P* values are included within each panel. Sample sizes were determined on the basis of previous experiments using similar methodologies. All depicted data points are biological replicates taken from distinct samples. Each figure consists of a minimum of three independent experiments from multiple biological replicates, unless stated otherwise in the figure legends. *n* refers to the number of animals or the number of independent experiments with cell lines. For in vivo studies, age-matched mice were randomly assigned to treatment groups.
