# Supplementary material for: Mitochondrial respiratory complex III sustains IL-10 production in activated macrophages and promotes tumor-mediated immune evasion
Source: Sci Adv. 2025 Jan 22;11(4):eadq7307. doi: 10.1126/sciadv.adq7307 (PMC11789823; doi:10.1126/sciadv.adq7307)
Supplement: Supplementary file 1 — Figs. S1 to S11 [file sciadv.adq7307_sm.pdf]

Science Advances
AAAS

Supplementary Materials for

Mitochondrial respiratory complex III sustains IL-10 production in activated macrophages and promotes tumor-mediated immune evasion

Alessia Zotta *et al.*

Corresponding author: Luke A. J. O'Neill, laoneill@tcd.ie

Sci. Adv. **11**, eadq7307 (2025)
DOI: 10.1126/sciadv.adq7307

This PDF file includes:

Figs. S1 to S11

Supplementary Figures

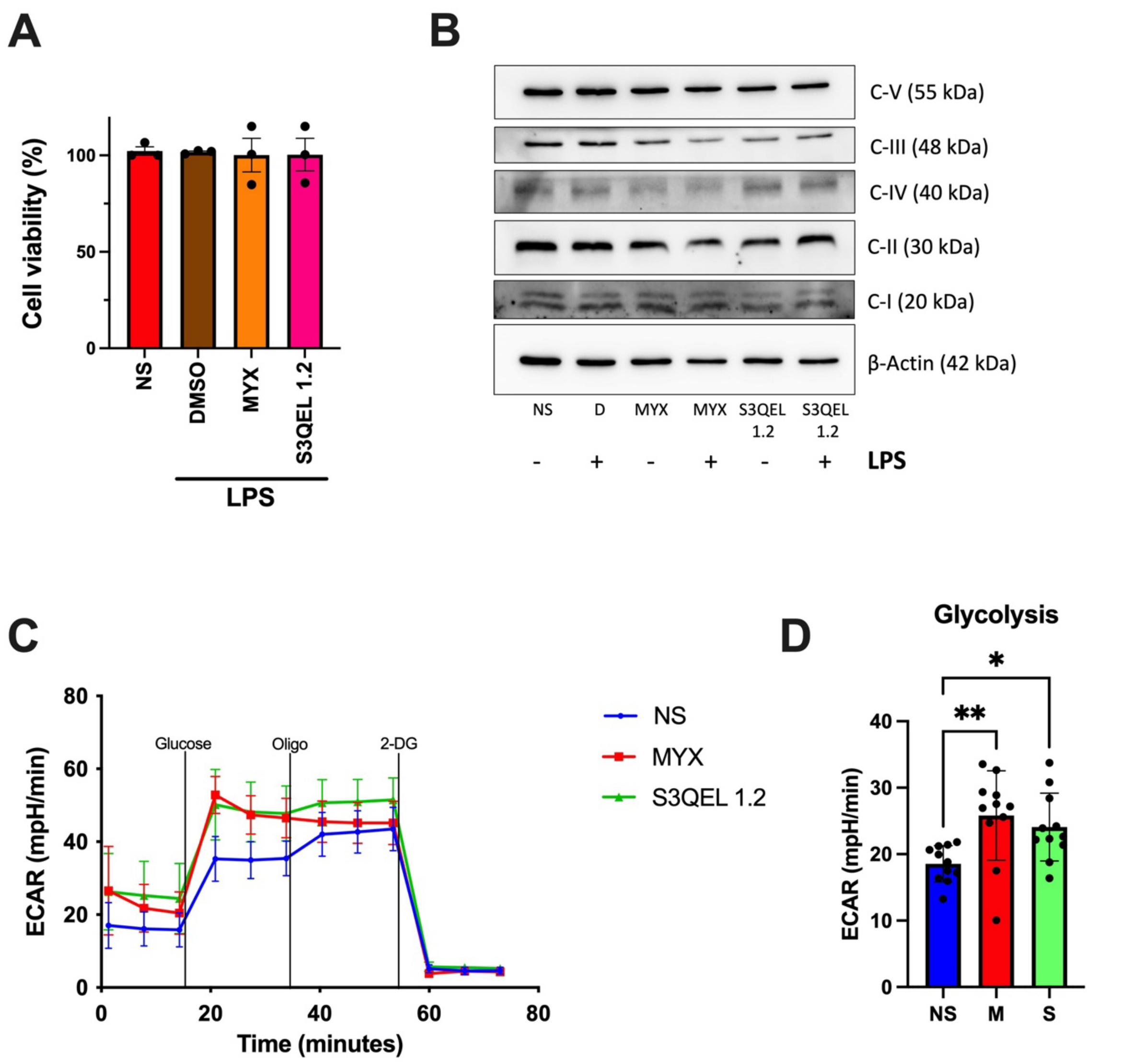

Supplementary Fig. 1. S3QEL and MYX effects on macrophage viability and metabolic activity.

(A-B) BMDMs were pre-treated with DMSO, MYX (500nM), or S3QEL 1.2 (10µM) (3 hrs) prior to LPS (100ng/mL) stimulation (4 hrs) and (**A**) cell viability was measured by reduced MTT (formazan crystals). Western blot **(B)** of mitochondrial C-I, C-II, C-III, C-IV and C-V in cell lysates, normalized to β-Actin. (**C-D)** BMDMs were treated for 3 hours with DMSO, S3QEL 1.2 (10µM) and MYX (500nM). Real-time changes in ECAR were measured by Seahorse XF analysis. (**C)** Representative

ECAR trace from one of three independent experiments, each experiment being performed with 11 technical replicates. Glycolysis (**D)** rate was calculated and displayed as bar charts. Data are mean ± SD from one independent experiment. Data from (**A**) are mean ± SEM from 3 independent experiments. Data from (**B**) are mean ± SEM from 3 independent experiments, bands are representative of one independent experiment. Data from (**C-D**) are mean ± SD from one independent experiment, n=11 technical replicates for each condition. Western blot bands are from one representative experiment. Data from P values were calculated using one-way ANOVA for multiple comparisons. Differences were considered statistically significant at *p < 0.05, **p < 0.01, ***p < 0.001, ****p < 0.0001.

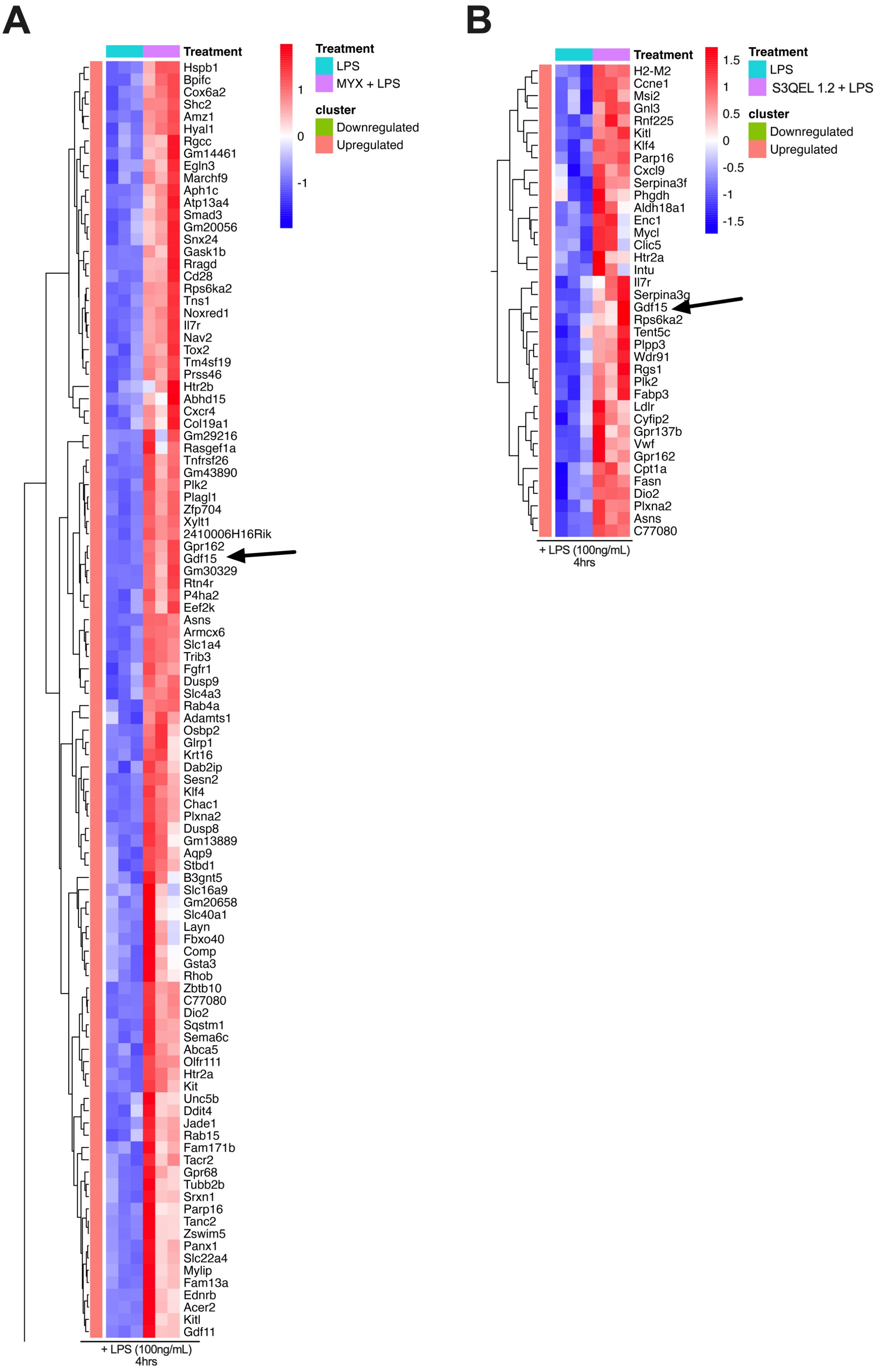

A
Treatment
LPS
MYX + LPS
cluster
Downregulated
Upregulated
1
0
-1
Hspb1
Bpifc
Cox6a2
Shc2
Amz1
Hyal1
Rgcc
Gm14461
Egln3
Marchf9
Aph1c
Atp13a4
Smad3
Gm20056
Snx24
Gask1b
Rragd
Cd28
Rps6ka2
Tns1
Noxred1
Il7r
Nav2
Tox2
Tm4sf19
Prss46
Htr2b
Abhd15
Cxcr4
Col19a1
Gm29216
Rasgef1a
Tnfrsf26
Gm43890
Plk2
Plagl1
Zfp704
Xylt1
2410006H16Rik
Gpr162
Gdf15
Gm30329
Rtn4r
P4ha2
Eef2k
Asns
Armcx6
Slc1a4
Trib3
Fgfr1
Dusp9
Slc4a3
Rab4a
Adamts1
Osbp2
Glrp1
Krt16
Dab2ip
Sesn2
Klf4
Chac1
Plxna2
Dusp8
Gm13889
Aqp9
Stbd1
B3gnt5
Slc16a9
Gm20658
Slc40a1
Layn
Fbxo40
Comp
Gsta3
Rhob
Zbtb10
C77080
Dio2
Sqstm1
Sema6c
Abca5
Olfr111
Htr2a
Kit
Unc5b
Ddit4
Jade1
Rab15
Fam171b
Tacr2
Gpr68
Tubb2b
Srxn1
Parp16
Tanc2
Zswim5
Panx1
Slc22a4
Mylip
Fam13a
Ednrb
Acer2
Kitl
Gdf11
+ LPS (100ng/mL)
4hrs
B
Treatment
LPS
S3QEL 1.2 + LPS
cluster
Downregulated
Upregulated
1.5
1
0.5
0
-0.5
-1
-1.5
H2-M2
Ccne1
Msi2
Gnl3
Rnf225
Kitl
Klf4
Parp16
Cxcl9
Serpina3f
Phgdh
Aldh18a1
Enc1
Mycl
Clic5
Htr2a
Intu
Il7r
Serpina3g
Gdf15
Rps6ka2
Tent5c
Plpp3
Wdr91
Rgs1
Plk2
Fabp3
Ldlr
Cyfip2
Gpr137b
Vwf
Gpr162
Cpt1a
Fasn
Dio2
Plxna2
Asns
C77080
+ LPS (100ng/mL)
4hrs

Supplementary Fig. 2. MYX and S3QEL 1.2 differentially upregulate specific genes together with LPS stimulation.

BMDMs were treated for 3 hours with DMSO, S3QEL 1.2 (10µM) and Myxothiazol (500nM) and then stimulated for 4 hours with LPS (100ng/mL). After that, cell lysates were harvested, quantified and RNAseq was performed. **(A)** Heatmap of significantly upregulated genes by MYX with adjusted p value<0.1. **(B)** Heatmap of significantly upregulated genes by S3QEL 1.2 with adjusted p value<0.1. Normalized counts were converted to Z-score [(normalized counts-mean)/StDev]. Data are from n=3 from one independent experiment; LPS 4h

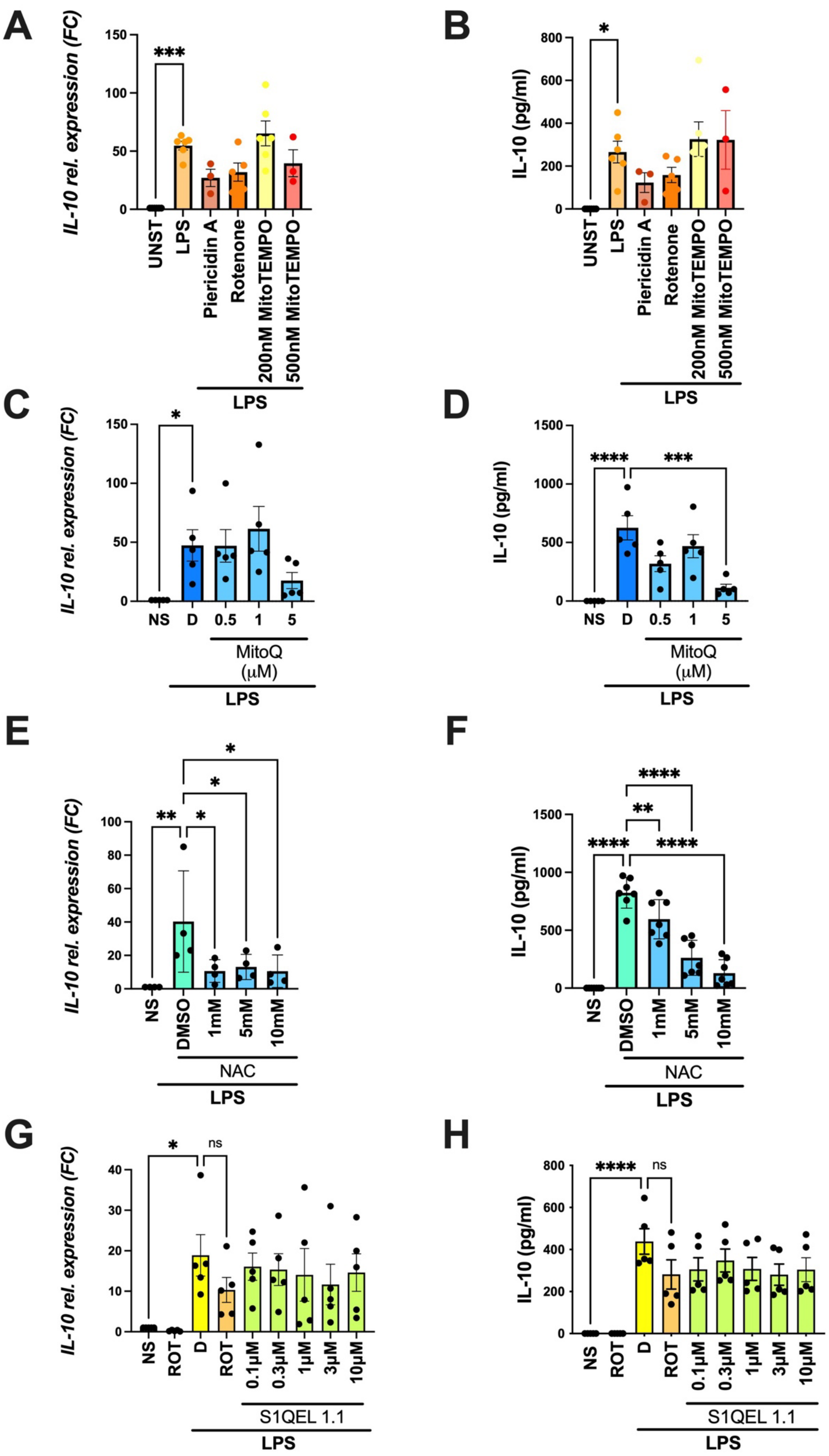
A
IL-10 rel. expression (FC)
***
UNST
LPS
Piericidin A
Rotenone
200nM MitoTEMPO
500nM MitoTEMPO
LPS
B
IL-10 (pg/ml)
*
UNST
LPS
Piericidin A
Rotenone
200nM MitoTEMPO
500nM MitoTEMPO
LPS
C
IL-10 rel. expression (FC)
*
NS
D
0.5
1
5
MitoQ (μM)
LPS
D
IL-10 (pg/ml)
****
***
NS
D
0.5
1
5
MitoQ (μM)
LPS
E
IL-10 rel. expression (FC)
**
*
*
*
NS
DMSO
1mM
5mM
10mM
NAC
LPS
F
IL-10 (pg/ml)
****
**
****
****
NS
DMSO
1mM
5mM
10mM
NAC
LPS
G
IL-10 rel. expression (FC)
*
ns
NS
ROT
D
ROT
0.1μM
0.3μM
1μM
3μM
10μM
S1QEL 1.1
LPS
H
IL-10 (pg/ml)
****
ns
NS
ROT
D
ROT
0.1μM
0.3μM
1μM
3μM
10μM
S1QEL 1.1
LPS

Supplementary Figure 3. IL-10 decrease is redox sensitive and Complex I-independent.

(A-B) BMDMs were pre-treated with DMSO, ROT (5µM), Piericidin A (100 nM) or MitoTEMPO (200nM or 500nM) for 1 hr prior to LPS (100ng/mL) stimulation for 4 hrs and cell lysates and supernatants were harvested. Quantification of *IL-10* (**A**) mRNA by RT-qPCR, relative to *Rps18* housekeeping gene. (**B**) Quantification of IL-10 levels by ELISA. mRNA by RT-qPCR, relative to *Rps18* housekeeping gene. **(C-D)** BMDMs were pre-treated with DMSO, MitoQ (0,5, 1, 5µM) for 1 hr prior to LPS (100ng/mL) stimulation for 4 hrs and cell lysates and supernatants were harvested. Quantification of *IL-10* (**C**) mRNA by RT-qPCR, relative to *Rps18* housekeeping gene. (**D**) Quantification of IL-10 levels by ELISA. (**E-F)** BMDMs were pre-treated with NAC (1mM-10mM) for 1 hour prior to LPS (100ng/mL) stimulation for 4 hrs and cell lysates and supernatants were harvested. Quantification of *IL-10* (**E**) mRNA by RT-qPCR, relative to *Rps18* housekeeping gene. (**F**) Quantification of IL-10 levels by ELISA. **(G-H)** BMDMs were pre-treated with DMSO, ROT (5µM) or S1QEL 1.1 (0,1-10µM) for 3 hrs prior to LPS (100ng/mL) stimulation for 4 hrs and cell lysates and supernatants were harvested. Quantification of *IL-10* (**G**) mRNA by RT-qPCR, relative to *Rps18* housekeeping gene. (**H**) Quantification of IL-10 levels by ELISA. Data from (**A-B**) are mean ± SEM for n=3-5 from 3 independent experiments. Data from (**C-D**) are mean ± SEM for n=5 from three independent experiments. Data from (**E-F**) are mean ± SEM for n=4-7 from two or three independent experiments. Data from (**G-H**) are mean ± SEM for n=5 from 3 independent experiments. P values were calculated using one-way ANOVA for multiple comparisons. Differences were considered statistically significant at $^*p<0.05$, $^{***}p < 0.001$, $^{****}p < 0.0001$.

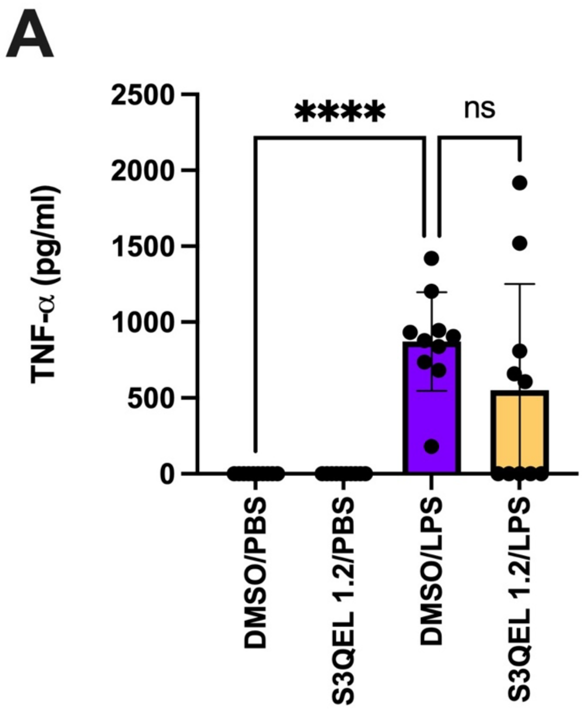

Supplementary Figure 4. TNF-α is not affected by S3QEL 1.2 *in vivo*.

Mice were intraperitoneally injected with PBS or S3QEL 1.2 (1mg/kg) for 2 hrs, followed by LPS (2,5 mg/kg) for 2 hrs. Blood (then serum) was collected. (**A)** Quantification of TNF-α by ELISA in the serum. Data from (**A**) are expressed as mean ± SEM (n=10 per group within two independent *in vivo* experiments). P values were calculated using one-way ANOVA for multiple comparisons. Differences were considered statistically significant at ****p < 0.0001.

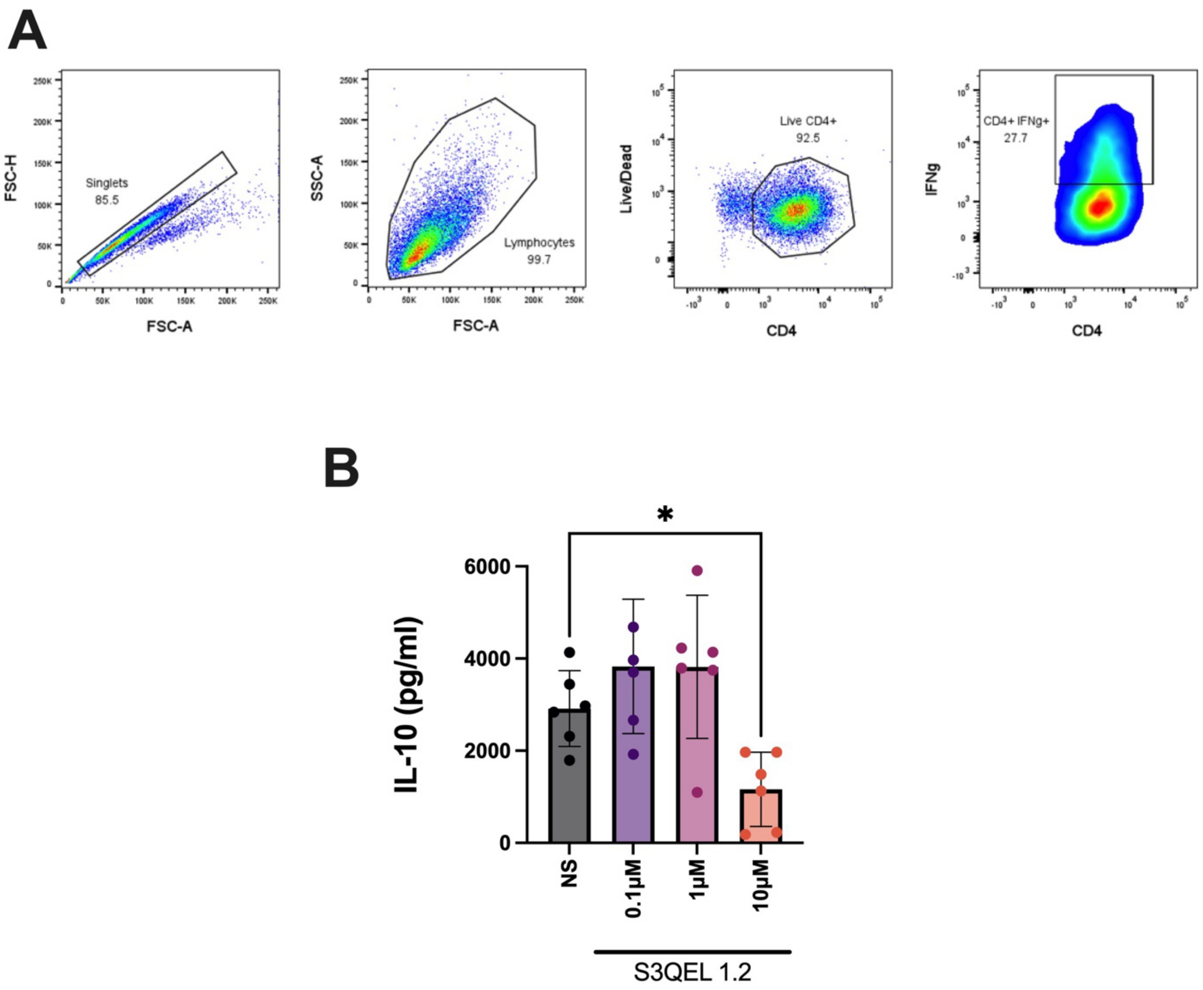

Supplementary Figure 5. S3QEL 1.2 decreases IL-10 release by Th1 cells. (**A-B**) Th1 cells were treated with DMSO or S3QEL 1.2 (0,1, 1 or 10 μM) for 72 hrs and, at the same time, stimulated with IL-2 and IL-12 and supernatants were harvested. (**A**) Gating strategy for identification of Th1 lymphocytes by flow cytometry. Representative contour plots of $CD4^+$ and $IFN\gamma^+$ population. (**B**) Quantification of IL-10 levels by ELISA. Data from (**B**) are mean ± SD from 2 independent experiments, each with 2-4 replicates. P values were calculated using one-way ANOVA for multiple comparisons. Differences were considered statistically significant at *p<0.05.

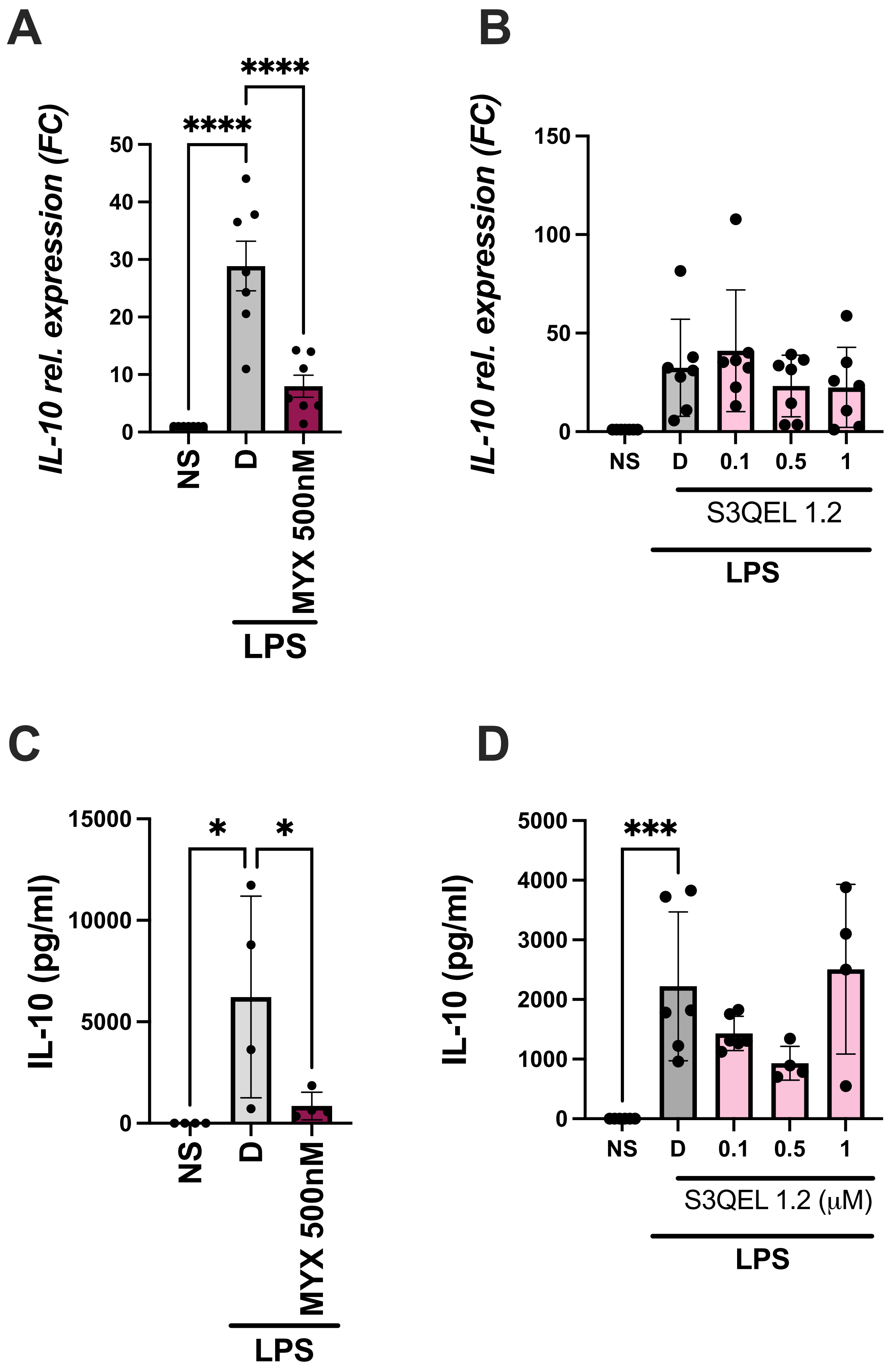
A
IL-10 rel. expression (FC)
0
10
20
30
40
50
****
****
NS
D
MYX 500nM
LPS
B
IL-10 rel. expression (FC)
0
50
100
150
NS
D
0.1
0.5
1
S3QEL 1.2
LPS
C
IL-10 (pg/ml)
0
5000
10000
15000
*
*
NS
D
MYX 500nM
LPS
D
IL-10 (pg/ml)
0
1000
2000
3000
4000
5000
***
NS
D
0.1
0.5
1
S3QEL 1.2 (μM)
LPS

Supplementary Figure 6. S3QEL 1.2 and MYX pre-treatment of LPS-stimulated human macrophages.

(A-D) Macrophages differentiated from human PBMCs with hM-CSF were pre-treated with DMSO, S3QEL 1.2 (10µM) or MYX (500nM) for 3 hrs prior to LPS (100ng/mL) stimulation for 24 hrs and cell lysates and supernatants were harvested. Quantification of *IL-10* (**A-B**) mRNA by RT-qPCR, relative to *Rps13* housekeeping gene. (**C-D**) Quantification of IL-10 levels by ELISA. Data from (**A-B**) are mean ± SEM for n=7 from 3 three independent experiments. Data from (**C-D**) are mean ± SEM for n=4-6 from three independent experiment. P values were calculated using one-way ANOVA for multiple comparisons. Differences were considered statistically significant at *p<0.05, ***p < 0.001, ****p < 0.0001.

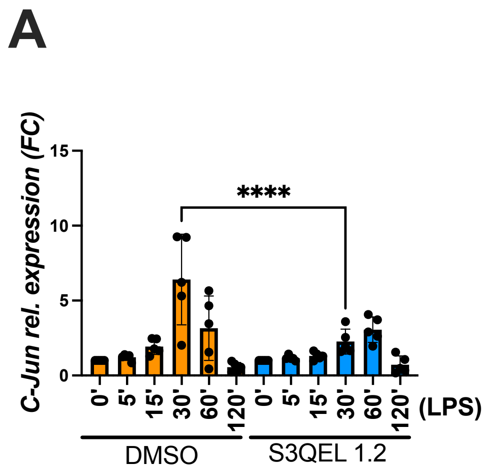

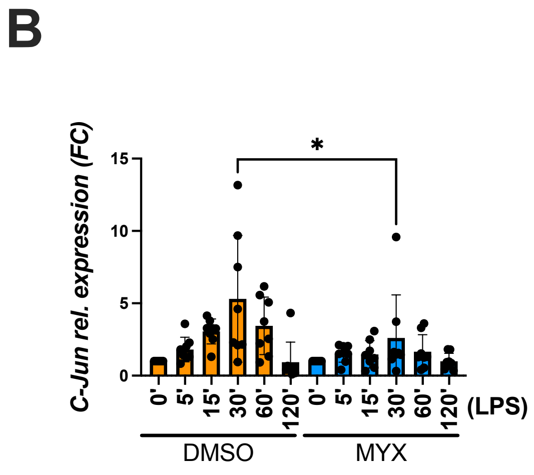

Supplementary Figure 7. S3QEL 1.2 and MYX effects on c-Jun.

(A-B) BMDMs were pre-treated with DMSO, MYX (500nM) or S3QEL 1.2 (10µM) for 1 hour prior to LPS (100ng/mL) stimulation for 0-120 minutes and cell lysates were harvested. Quantification of c-*Jun* mRNA by RT-qPCR, relative to *Rps18* housekeeping gene, in BMDMs pretreated with S3QEL 1.2 (**A**) or MYX (**B**) and stimulated with LPS. Data from (**A and B**) are mean ± SEM from n=5-7 from 3 independent experiments. P values were calculated using one-way ANOVA for multiple comparisons. Differences were considered statistically significant at $^*p < 0.05$, $^{****}p < 0.0001$.

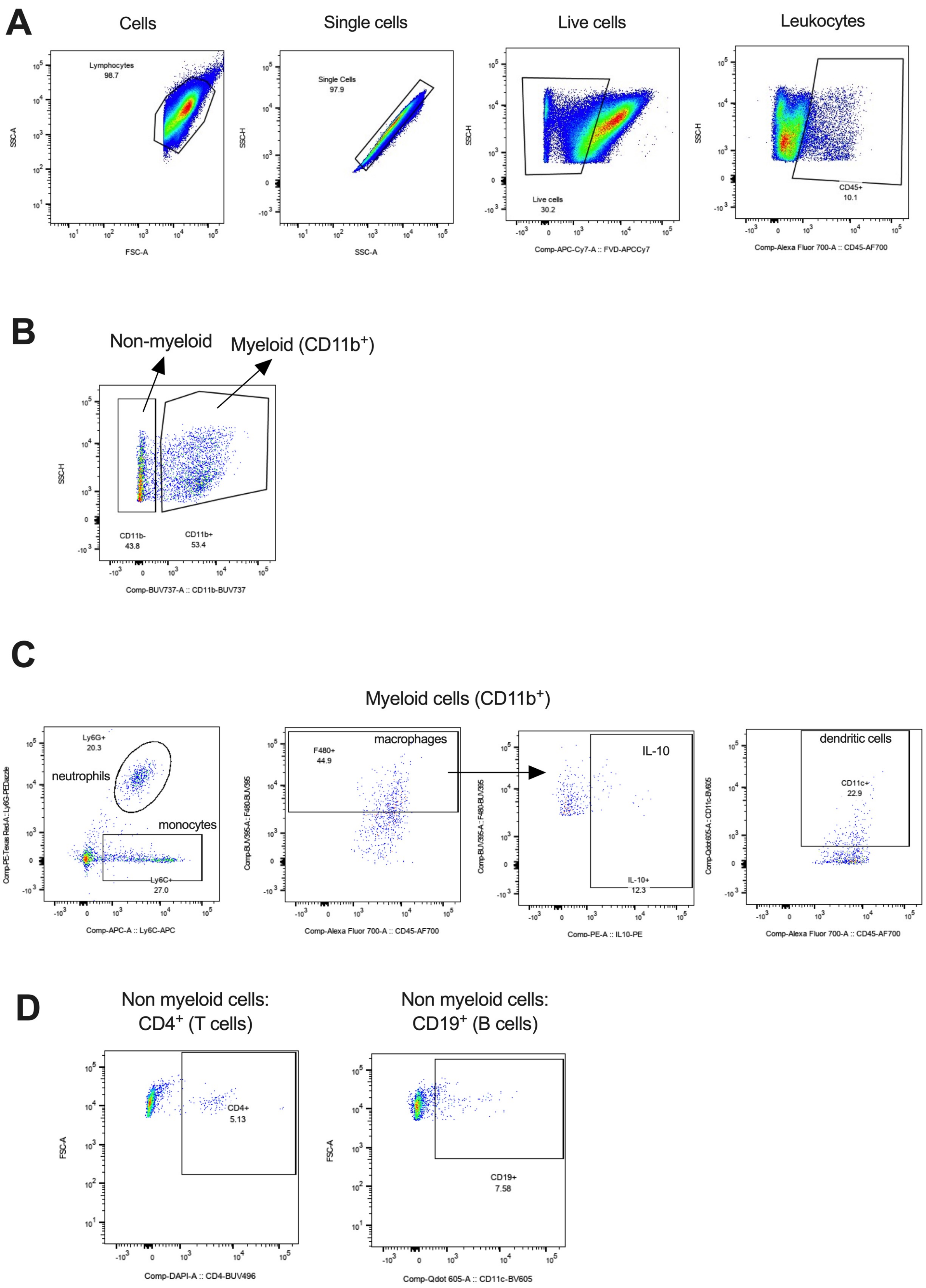
A
Cells
Lymphocytes
98.7
SSC-A
FSC-A
Single cells
Single Cells
97.9
SSC-H
SSC-A
Live cells
Live cells
30.2
SSC-H
Comp-APC-Cy7-A :: FVD-APCCy7
Leukocytes
CD45+
10.1
SSC-H
Comp-Alexa Fluor 700-A :: CD45-AF700
B
Non-myeloid
Myeloid (CD11b+)
CD11b-
43.8
CD11b+
53.4
SSC-H
Comp-BUV737-A :: CD11b-BUV737
C
Myeloid cells (CD11b+)
Ly6G+
20.3
neutrophils
monocytes
Ly6C+
27.0
Comp-APC-A :: Ly6C-APC
F480+
44.9
macrophages
Comp-Alexa Fluor 700-A :: CD45-AF700
IL-10
IL-10+
12.3
Comp-PE-A :: IL10-PE
dendritic cells
CD11c+
22.9
Comp-Alexa Fluor 700-A :: CD45-AF700
D
Non myeloid cells:
CD4+ (T cells)
CD4+
5.13
FSC-A
Comp-DAPI-A :: CD4-BUV496
Non myeloid cells:
CD19+ (B cells)
CD19+
7.58
FSC-A
Comp-Qdot 605-A :: CD11c-BV605

Supplementary Fig 8. Gating strategy for myeloid and non-myeloid cell subsets isolated from B16F10 tumor mass.

(**A**) Gating strategy for identification of leukocytes by flow cytometry. (**B**) Representative contour plots of $CD11b^-$ (non-myeloid) and $CD11b^+$ (myeloid) cells. (**C**) Gating strategy for $Ly6G^+$ (neutrophils), $Ly6C^+$ (monocytes), IL-10 secretion and $CD11c^+$ (dendritic cells) cells. (**D**) Representative contour plots showing $CD4^+$ (non-myeloid cells, T cells) and $CD19^+$ (non-myeloid cells, B cells). IL-10 not detected in lymphocytes due to our specific readouts focused on macrophages/dendritic cells.

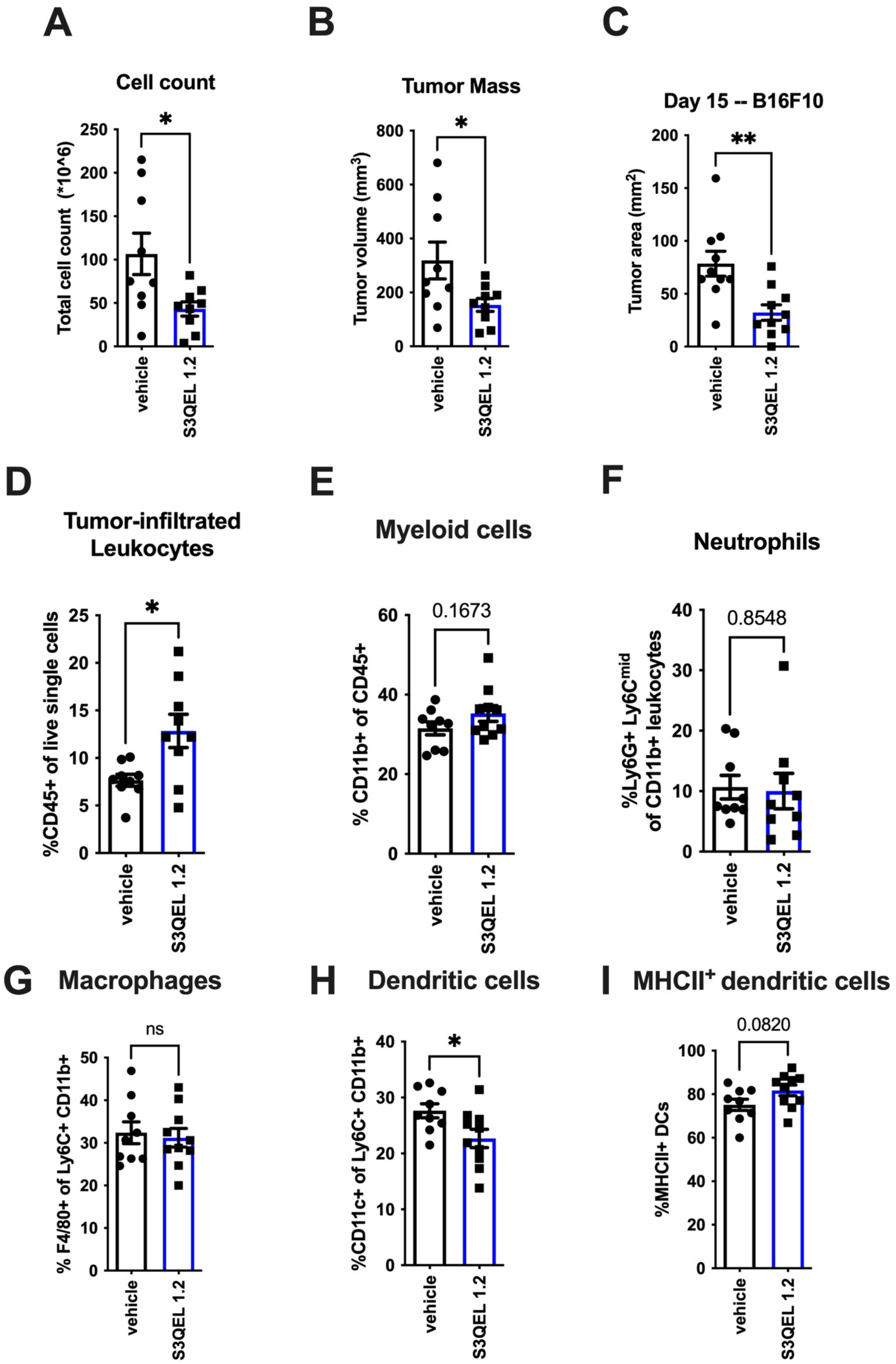

A
Cell count
Total cell count (*10^6)
B
Tumor Mass
Tumor volume (mm3)
C
Day 15 -- B16F10
Tumor area (mm2)
D
Tumor-infiltrated Leukocytes
%CD45+ of live single cells
E
Myeloid cells
0.1673
% CD11b+ of CD45+
F
Neutrophils
0.8548
%Ly6G+ Ly6Cmid of CD11b+ leukocytes
G
Macrophages
ns
% F4/80+ of Ly6C+ CD11b+
H
Dendritic cells
%CD11c+ of Ly6C+ CD11b+
I
MHCII+ dendritic cells
0.0820
%MHCII+ DCs
vehicle
S3QEL 1.2

Supplementary Fig. 9. Analysis of immune cells infiltrating the B16F10 melanoma *ex vivo.*

Mice were challenged with $5x10^6$ B16F10 cells and administered with vehicle or S3QEL 1.2 (1mg/kg). Tumors were measured daily. (**A**) Total cell counts from tumors collected at the endpoint of the experiment. Tumor mass (**B**) and tumor volume (**C**) assessed at the endpoint are shown. (**D-I)** Immune cells infiltrating the tumor were analyzed by flow cytometry *ex vivo*. (**D**) Flow cytometry data for cells expressing the surface marker CD45 showing percentage of $CD45^+$ cells. (**E**) Flow cytometry data for cells expressing the surface marker CD11b showing percentage of $CD11b^+$ cells. (**F**) Flow cytometry data for cells expressing the surface markers Ly6G and Ly6C showing percentage of $Ly6G^+Ly6C^{mid}$ cells. (**G-I**) Flow cytometry data showing percentage of $F4/80^+Ly6C^+CD11b^+$ cells (**G**) and $CD11b^+CD11c^+Ly6C^+$ cells (**H**). (**I**) Flow cytometry data showing percentage of $MHCII^+$ dendritic cells. Data from (**A-I**) are mean ± SD for n=9 from one independent *in vivo* experiment. P values were calculated using two-tailed unpaired Student's T test. Differences were considered statistically significant at *p < 0.05, **p < 0.01, ***p < 0.001, ****p < 0.001.

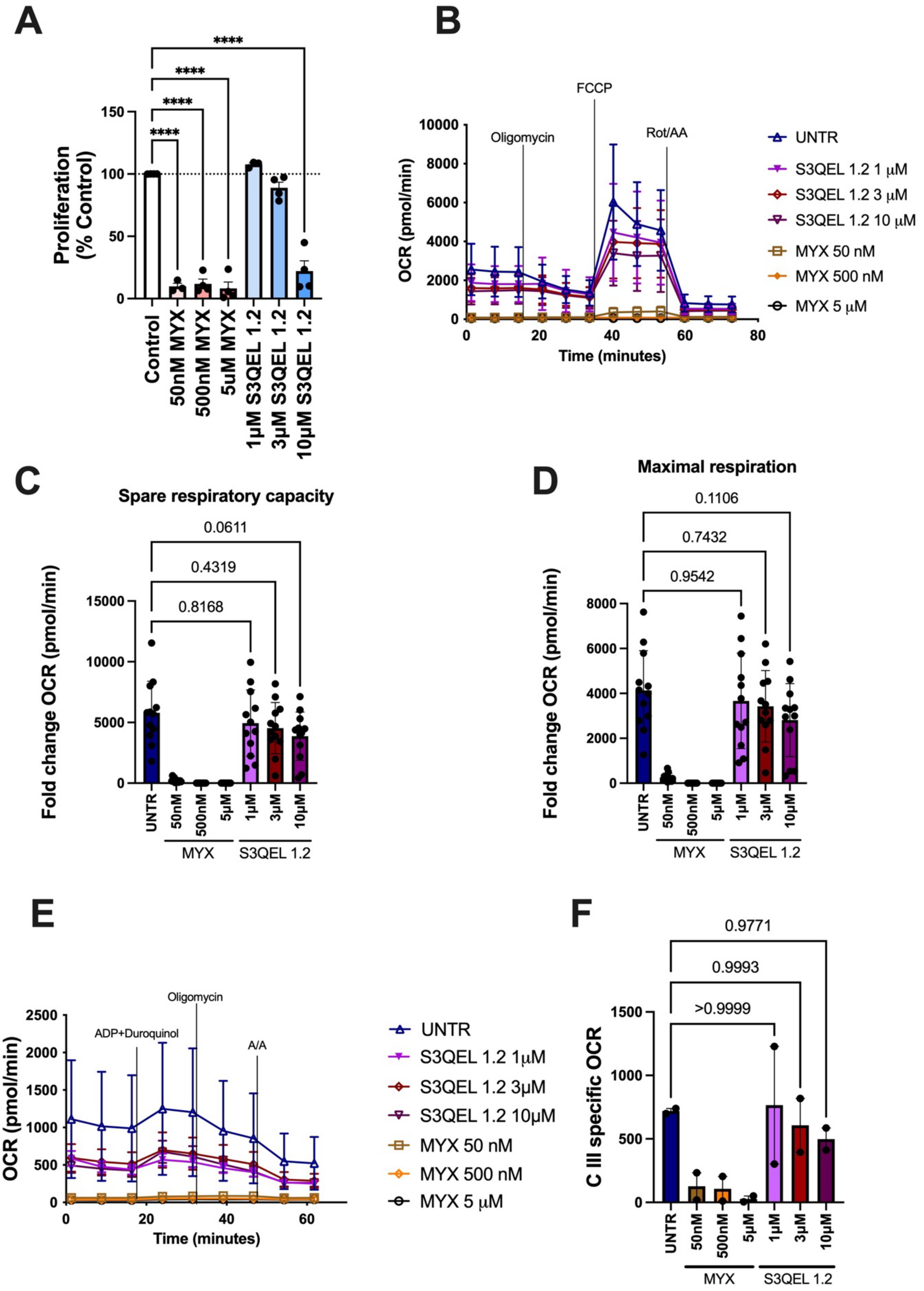

A
****
****
****
****
Proliferation (% Control)
150
100
50
0
Control
50nM MYX
500nM MYX
5uM MYX
1μM S3QEL 1.2
3μM S3QEL 1.2
10μM S3QEL 1.2
B
FCCP
Oligomycin
Rot/AA
OCR (pmol/min)
10000
8000
6000
4000
2000
0
0
20
40
60
80
Time (minutes)
UNTR
S3QEL 1.2 1 μM
S3QEL 1.2 3 μM
S3QEL 1.2 10 μM
MYX 50 nM
MYX 500 nM
MYX 5 μM
C
Spare respiratory capacity
0.0611
0.4319
0.8168
Fold change OCR (pmol/min)
15000
10000
5000
0
UNTR
50nM
500nM
5μM
1μM
3μM
10μM
MYX
S3QEL 1.2
D
Maximal respiration
0.1106
0.7432
0.9542
Fold change OCR (pmol/min)
8000
6000
4000
2000
0
UNTR
50nM
500nM
5μM
1μM
3μM
10μM
MYX
S3QEL 1.2
E
Oligomycin
ADP+Duroquinol
A/A
OCR (pmol/min)
2500
2000
1500
1000
500
0
0
20
40
60
Time (minutes)
UNTR
S3QEL 1.2 1μM
S3QEL 1.2 3μM
S3QEL 1.2 10μM
MYX 50 nM
MYX 500 nM
MYX 5 μM
F
0.9771
0.9993
>0.9999
C III specific OCR
1500
1000
500
0
UNTR
50nM
500nM
5μM
1μM
3μM
10μM
MYX
S3QEL 1.2

Supplementary Fig. 10. Effects of S3QEL 1.2 and MYX on melanoma cells growth and respiration.

(A) B16F10 melanoma cells were treated with DMSO, MYX (50nM, 500nM, 5μM) or S3QEL 1.2 (1, 3 and 10μM) and allowed proliferating for 2-3 days. Quantification of cells coloured with crystal violet dye. **(B-D)** B16F10 melanoma cells were treated with DMSO, MYX (50nM, 500nM, 5μM) or S3QEL 1.2 (1, 3 and 10μM) for 3 hrs. Seahorse Mito Stress Test showing oxygen consumption rate (OCR) of cells over time after addition of oligomycin (oligo), carbonyl cyanide-4-phenylhydrazone (FCCP) and rotenone/antimycin A (Rot/AA) **(B)**. Maximal respiration **(C)** and spare respiratory capacity **(D)** as calculated from the OCR values. **(E-F)** B16F10 melanoma cells were treated with DMSO, MYX (50nM, 500nM, 5μM) or S3QEL 1.2 (1, 3 and 10μM) for 3 hrs. **(E)** Seahorse Extracellular Flux Analysis showing C-III-specific-oxygen consumption rate (OCR) of cells over time after addition of ADP + Duroquinol, oligomycin and antimycin A (AA). **(F)** Quantification of C-III specific-OCR as calculated from the OCR values. Data in **(A)** are expressed as mean ± SEM (n=4-7 technical replicates from three independent experiments). Data from (**B-D**) are mean ± SEM from 2 independent experiments, n=12 technical replicates for each condition. Data from (**E-F**) are mean ± SEM from 2 independent experiments, n=12 technical replicates for each condition. ****$p<0.0001$ as calculated using a one-way ANOVA followed by Sidak's multiple comparisons test.

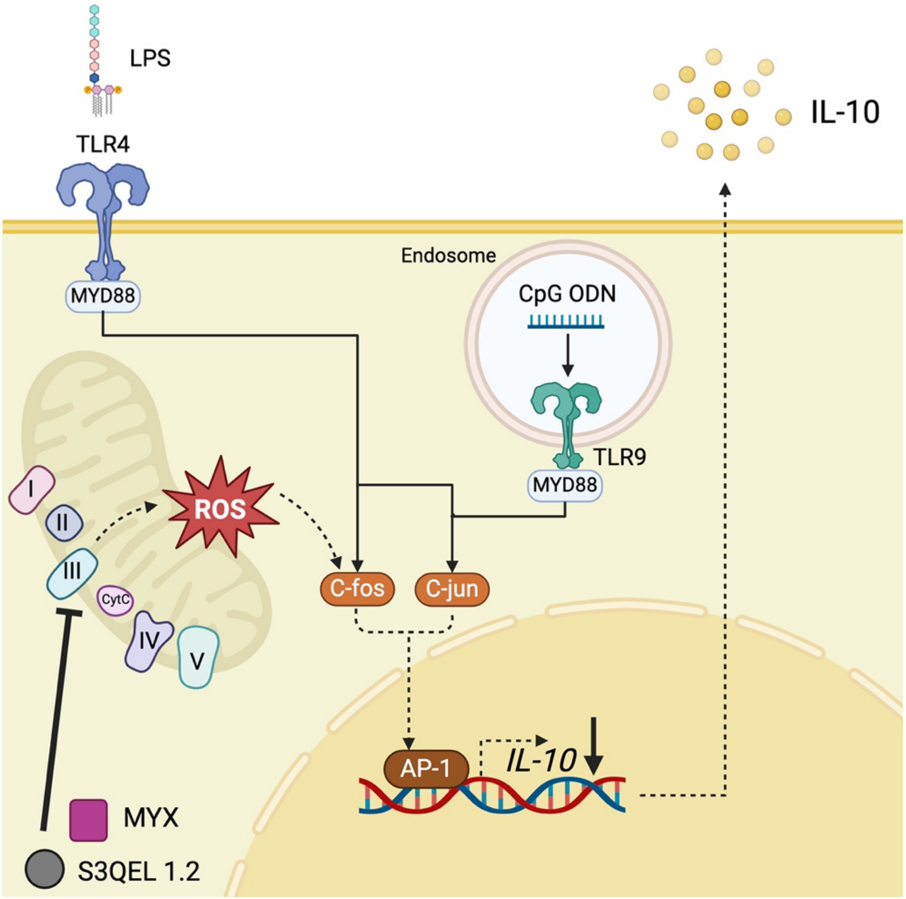

Supplementary Fig. 11. LPS and CpG-induced IL-10 decrease is dependent on mitochondrial respiratory Complex III and is mediated by AP-1 suppression.

MYX and S3QEL 1.2 inhibit ROS generation from complex III, repressing IL-10 production in macrophages activated by LPS and CpG. MYX and S3QEL 1.2 decrease the protein levels of c-fos and c-jun, which together dimerize to form the transcription factor AP-1. In the presence of Complex III inhibitors, AP-1 does not translocate to the nucleus, and it is unable to increase IL-10 transcription.

MYX: myxothiazol
S3QEL: S3QEL 1.2
ROS: reactive oxygen species
IL-10: interleukin 10
LPS: lipopolysaccharide
CpG (ODN): CpG oligodeoxynucleotides
AP-1: Activator protein 1
